# Phyllite/bentonite mixture—an alternative effective buffer material for a geological disposal of radioactive waste

**DOI:** 10.1007/s11356-023-31102-6

**Published:** 2023-12-08

**Authors:** Joanna Kyzioł-Komosińska, Janusz Janeczek, Agnieszka Dzieniszewska, Monika J. Fabiańska, Aniela Matuszewska, Ewa Teper, Ewa Szram, Tomasz Krzykawski, Magdalena Pająk, Justyna Czupiol

**Affiliations:** 1 Institute of Environmental Engineering, Polish Academy of Sciences, 34 M Skłodowskiej-Curie St., 41-819 Zabrze, Poland; 2 Institute of Earth Sciences, University of Silesia, 60 Będzińska St., 41-200 Sosnowiec, Poland

**Keywords:** Phyllite/bentonite mixtures, Europium(III) ions, Adsorption/desorption, Radioactive waste repository barriers

## Abstract

The use of phyllite (Phy) instead of quartz in mixtures with bentonite (B) is recommended as a buffer material for engineering barriers in a geological repository of nuclear waste. The recommendation is based on experimentally determined sorption properties of various Phy/B mixtures. The adsorption capacity of Phy/B mixtures (Phy/B: 75/25, 50/50, and 25/75), the removal efficacy of Eu(III) ions (an analog for fissiongenic lanthanides and actinides), and the rate of their binding reaction were studied using the batch adsorption equilibrium and kinetic experiments at different Eu(III) initial concentrations, solution pH, and solution to adsorbent (L/S) ratio. The adsorption capacity of the Phy/B mixtures increased with the increased bentonite content in the mixture depending on the L/S ratio and solution pH. The highest increase in the adsorption capacity of the Phy/B mixtures compared to phyllite was observed for the Phy/B proportions of 25/75 and 50/50. The rate of the Eu(III) adsorption was the best fitted by the pseudo-second-order kinetic model indicating that the adsorption rate was controlled by chemisorption. The Sips model provided the best correlation of the adsorption experimental data, indicative of more than one adsorption site. The results of this study show the advantage of the Phy/B mixtures in immobilizing Eu and certain fission products by combining adsorption properties of the materials.

**Supplementary Information:**

The online version contains supplementary material available at 10.1007/s11356-023-31102-6.

## Introduction

One of the specific goals of radioactive waste disposal is to “inhibit, reduce, and delay the migration of radionuclides at any time from the waste to the accessible biosphere” (IAEA Safety Standards 2011). To achieve this goal, the concept of a multibarrier disposal system in an underground (geological) repository has been developed. The system is made up of engineered and geological (host rock) barriers. The engineered barriers comprise the packaging with vitrified high-level nuclear waste (HLNW) or spent nuclear fuel (SNF), the clay barrier (buffor), and the backfill (Kale and Ravi 2021). The long-term safety of a geological repository for nuclear waste relies on the performance of each of these barriers. However, the role of the clay barrier is crucial in the repository safety assessment (e.g., Itälä 2009; Yang et al. 2014). The principal function of a clay barrier material around waste packages is to protect them from the intrusion of groundwater and, if such an event occurred, to prevent or limit the migration of released radionuclides.

One of the possible scenarios considered in the long-term performance assessment of a radioactive waste repository is the release of radionuclides from breached waste containers and their migration into groundwater through the barriers. The distribution and mobility of the released radionuclides are controlled by the adsorption and ion exchangeable properties of the clay barrier and backfill materials. Moreover, adsorption is important in retarding the potential migration of radionuclides to the aquatic systems and biosphere. Understanding the kinetic process and removal efficacy/adsorption capacity is fundamental for the prediction of radionuclide migration in the surroundings of deep geological disposal sites of high-level nuclear wastes. The studies mainly concern the adsorption capacity of radionuclides on pure minerals or their modified forms as well as on composite materials (Bradbury and Baeyens 2005; García et al. 2019; Hanza et al. 2018; Sharma and Tomar 2011; Verma et al. 2019; Virtanen et al. 2018). To design a highly effective geochemical reactive barrier to immobilize radionuclides in a waste repository, different materials must be parallel studied to analyze their adsorption capacity and adsorption kinetics. However, there have been only a few studies on the adsorption kinetics in this respect (e.g., Baumer and Hixon 2018; Wang 2018).

Currently, bentonite or bentonite mixed with quartz sand is considered to be used as a barrier and backfill material in the deep geological repository of HLNW and SNF because of their low permeability, plasticity (ability to swell), very good adsorption/ion exchange properties, and high thermal conductivity (Itälä 2009; Kónya et al. 2005; Pusch 1992, 2006; Sellin and Leupin 2014; Yang et al. 2014). However, bentonites undergo dehydration, mechanical degradation, and structural transformation decreasing their adsorption capacity at a temperature above 100 °C expected at the contact with waste containers, and other rocks of high thermal resistance and high adsorption capacity for radionuclides are being sought (Xiao et al. 2013). Previous studies show that argillaceous phyllites, while less efficient adsorbents than bentonites, have high adsorption capacity for low initial concentrations of actinides (< 5·10^−5^ M) and much higher adsorption capacity than quartz, especially at low pH (Kar et al. 2011; Kyzioł-Komosińska et al. 2019a). Moreover, they are thermally stable up to 400 °C. Therefore, argillaceous phyllite can be considered as a component of a mixture with bentonite instead of quartz to take advantage of phyllite adsorption capacity and proper mechanical properties (compensation for volume changes in swelling/shrinking bentonite), particularly at elevated temperatures. Phyllite clays have recently been used as barrier material in municipal waste landfills due to their low permeability (Garzón et al 2016).

The main purpose of this study is to determine the removal efficacy of Eu(III) ions by phyllite/bentonite (Phy/B) mixtures. Europium is an analog for fissiongenic lanthanides and actinides: Am and Cm; present in HLNW and SNF (NRC Regulation Part 63 2016; Sun et al 2020). Various Phy/B proportions were used in the adsorption experiments to determine the most efficacious mixture for immobilization Eu(III) ions. The rate of the Eu(III) ion removal from solution, the adsorption capacity of the Phy/B mixtures, and mechanisms of adsorption were investigated.

## Experimental

### Adsorbents

Phyllite used in this study was from the Dewon-Pokrzywna phyllite deposit in the northern foothills of the Opava Mountains in the Eastern Polish Sudetes (Sawicka et al. 2018). Bentonite was from the Miocene Kopernica deposit in Slovakia (Górniak et al. 2016). Mineral and chemical compositions together with textural and physical properties of these minerals are given in Kyzioł-Komosińska et al. (2019a). Powdered phyllite (Phy) and bentonite (B), both with particle size < 10 µm, were thoroughly mixed in weight proportions of 75/25, 50/50, and 25/75. The properties of phyllite, bentonite, and their mixtures relevant for the evaluation of their adsorption effectiveness are given in Table 1.

**Table 1:** Physicochemical properties of phyllite (Phy), bentonite (B), and the Phy/B mixtures

	Phy^a^	75Phy/25B^b^	50Phy/50B^b^	25Phy/75B^b^	B^a^
Specific surface area, m^2^/g	3.64	18.55	31.24	44.82	58.24
Cation exchange capacity, cmol_+_/kg	3.55	21.61	42.11	60.44	79.55
pH	6.85	6.87	6.97	7.15	7.41
pH_PZC_	6.21	6.14	6.08	6.01	5.95

^a^Kyzioł-Komosińska et al. (2019a)

^b^Kyzioł-Komosińska et al. (2019b)

### Adsorption experiments

The batch equilibration method was applied to examine the adsorption of Eu(III) from EuCl_3_·6H_2_O solution by the Ph/B mixtures under atmospheric pressure and at room temperature (23 ± 2 °C). Initial Eu(III) concentrations ranged from 0.01 mg/L (0.658·10^−7^ M) to ~ 200 mg/L (~ 1.316·10^−3^ M), and solution to adsorbent (L/S) ratio was 100:1 and 500:1. While in most disposal scenarios concentrations of released radionuclides are much lower than considered in this study (in the low ppm to ppb range), the wide range of initial Eu(III) concentrations used in the adsorption experiments allowed for the more precise measurements. Moreover, a high initial Eu(III) concentration may reflect the worst-case scenario of mass radionuclide release from the waste package after some catastrophic event.

Solution initial pH was adjusted to 4.5 and 7.0 by drop-wise addition of appropriate amounts of 0.01 M HCl or 0.01 M NaOH, respectively. The ionic strength was 0.010 M NaCl. The contact time of adsorbents and solutions was 360 min, i.e., more than necessary to achieve equilibrium inferred from the adsorption kinetics determination. The initial (*C*_0_) and equilibrium (*C*_*eq*_) concentrations of Eu(III) in supernatant solutions were measured after centrifugation and filtration either by the ICP-OES or by ICP-MS spectrometry depending on the Eu(III) concentrations. The removal efficacy (*RE*) of Eu(III) ions was calculated from the equation:

1 $$RE=\left[\frac{\left({C}_{0}-{C}_{eq}\right)}{{C}_{0}}\right]\cdot 100(\mathrm{\%})$$

Results obtained during this study allowed for the determination of the maximum initial concentrations (*C*_0*max*_) for which Eu(III) ions were fully adsorbed, i.e., *RE* = 99.9% and *C*_*eq*_ ≤ 0.001 mg/L.

Adsorption capacity (*q*) was calculated using the equation:

2 $$q=\frac{({C}_{0}-{C}_{eq})V}{m}(\mathrm{mg}/\mathrm{g})$$

where *m* mineral mass (g); *V* solution volume (L).

Duplicate samples were measured, and the standard error in the readings was less than 4%.

Two-parameter isotherm models of Freundlich (Freundlich 1906), Langmuir (Langmuir 1916), and Dubinin-Raduskevich (Dubinin 1960) and the three-parameter Sips isotherm model (Sips 1948) were used to estimate maximum adsorption capacity of the Phy/B mixtures and to determine the affinity between Eu(III) ions and adsorption centers in Phy and B and the bond energy (Table S1 in Supporting Information). The isotherm parameters were determined by non-linear regression using algorithms based on the Levenberg–Marquardt method (Statistic ver. 9.0 software). Two non-linear error functions (Foo and Hameed 2010) were examined in addition to the determination coefficient (*R*^*2*^) for fitting the calculated isotherm to the experimental data (Table S2 in Supporting Information). The correlation of experimental results to adsorption isotherms can help to understand the adsorption mechanisms and the degree of the heterogeneity of the adsorbent surface.

### Adsorption kinetics

To understand adsorption/desorption processes, both thermodynamic equilibrium and kinetics must be considered (Wang 2018). While thermodynamic data provide information only on the final state of a system, the study of kinetics reveals changes in chemical properties in time and is concerned with the rate of these changes (Azizian 2004).

The Phy/B mixtures were used in the batch kinetic experiments. The effect of the contact time was examined within 2.5–1440-min time-frame for *C*_0_ of 10 and 200 mg/L at pH 4.5 and 7.0 for L/S of 100:1. To determine the impact of the L:S on the adsorption rate, tests were carried out for L/S of 100:1 and 500:1 at *C*_0_ of 10 and 200 mg/L, and pH 7.0. Time elapsed to reach equilibrium in the solute-adsorbent system was further considered in the evaluation of the experimental results. Two kinetic-based reactions, i.e., Lagergren pseudo-first order (PFO) (Begg et al. 2018) and pseudo-second order (PSO) (Blanchard et al. 1984; Ho and McKay 1999), were applied to find the rate-determining step (Table S3 in Supporting Information). The kinetic parameters were estimated by non-linear regression. Tran et al. (2017) recommended the use of nonlinear forms of PFO and PSO to describe the kinetics of adsorption. Due to the transformation of nonlinear to linear forms, the units of the *Y* and *X* axes were changed. Two non-linear error functions (Table S2 in Supporting Information) were examined in addition to the coefficient (*R*^2^) to evaluate the fit of the equations to the experimental data (Foo and Hameed 2010).

### Auxilliary methods: scanning electron microscopy (SEM), attenuated total reflection Fourier transform infrared spectroscopy (ATR-FTIR)

Operating conditions for these methods are given in the Supporting Information.

## Results and discussion

### Adsorbents properties

The Phy/B mixtures differed significantly in physicochemical properties depending on the B content. The addition of 25% B into the Phy/B mixture caused a sixfold increase in CEC up to 21.6 cmol_+_/kg, i.e., to the value observed for kaolinite (Ma and Eggleton 1999). The CEC increased 12 times to 42.11 cmol_+_/kg in mixtures with 50% B. A similar effect of the B content was observed for the mixtures’ SSA (Table 1). Values of pH and pH_PZC_ of the Phy/B mixtures suspension increased from 6.87 to 7.15 and from 6.14 to 6.01, respectively, with an increase in B content.

### Europium precipitates

**Fig. 1:**
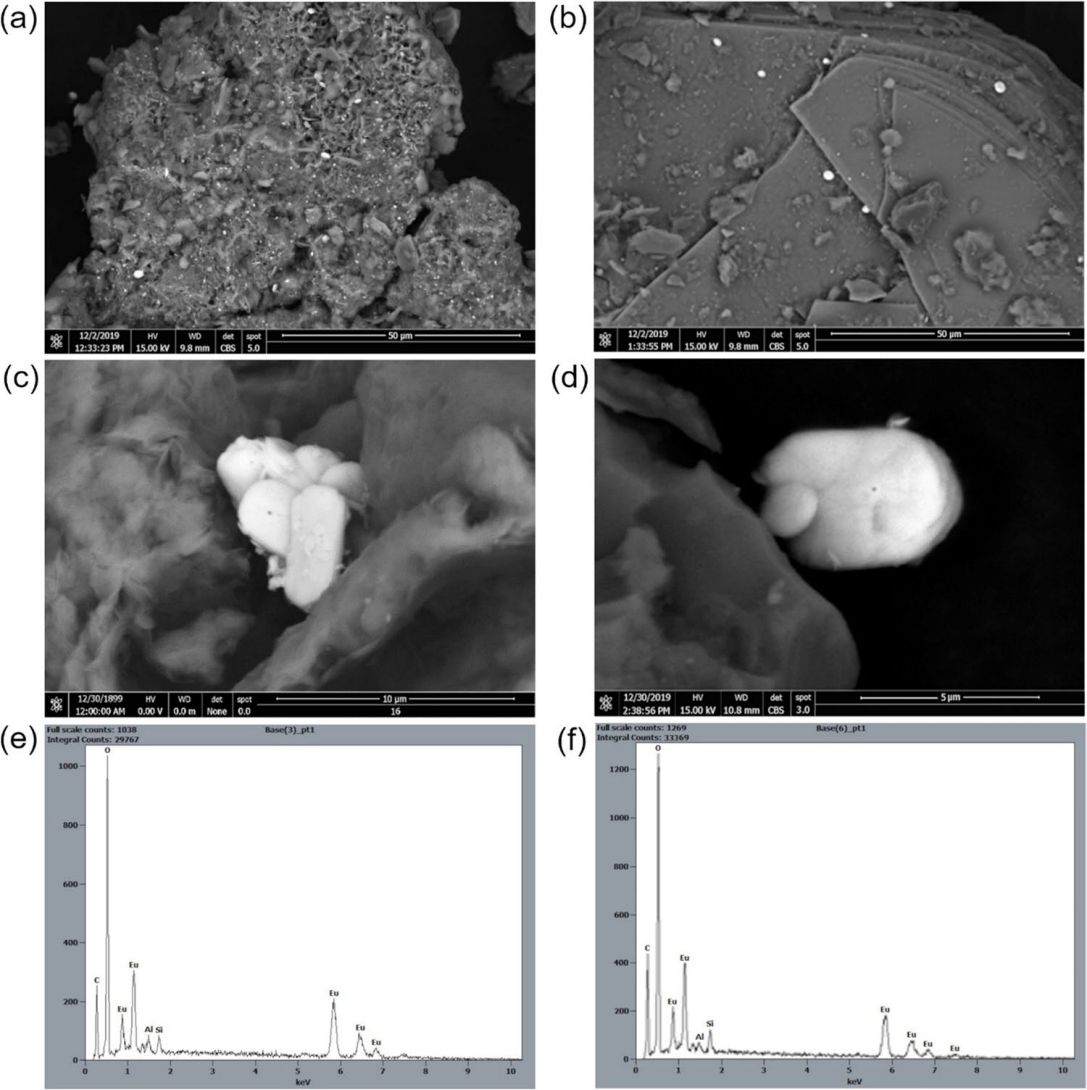
BSE images and EDS spectra of Eu precipitates on: **a** bentonite; **b** biotite partially replaced by Fe-chlorite in phyllite; close-ups (**c**, **d**) show platy crystals of Eu-phase with rounded edges; EDS spectra of the Eu-phase (**e**, **f**). Peaks of Al and Si are from the background minerals and C from the carbon-coating

Scanning electron microscopy (SEM)/EDS study of bentonite, phyllite, and the Phy/B mixtures (Table S4 in Supporting Information) after adsorption experiments revealed precipitates of the Eu-phase (Fig. 1). Elongated crystals of the Eu-phase were 5 μm long and < 4 μm wide. The rounded edges of those crystals suggest partial dissolution at the crystal-solution boundary layer. From semi-quantitative EDS analyses, the Eu contents in the crystals precipitated on bentonite and phyllite are on average 69.31 and 68.74 wt.%, respectively. These values are closer to the Eu(OH)_3_ stoichiometry (74.86 wt.% Eu) than to Eu_2_O_3_ (86.36 wt.% Eu) (Table S5 in Supporting Information).

The Eu precipitation was pH-dependent and occurred only at pH 7.0. The bulk contents of Eu adsorbed by montmorillonite in bentonite and by and chloritized biotite in phyllite were about 4.5 and 2.5 wt.%, respectively, regardless of solution pH.

### Infrared spectroscopy

Spectra of phyllite collected before and after Eu adsorption are not significantly different. The expected band shifts due to the formation of hydrogen bonds caused by the Eu(OH)^2+^ bound to –Al–OH were not observed in accord with suggestions by Bradbury and Baeyens (2005). The only significant change seen in the IR spectrum of phyllite after the adsorption experiment is the weakening of the 750 cm^−1^ band assigned to the SiAl–O–OH libration of chlorite hydroxyl sites.

FT-IR spectra of bentonite after Eu adsorption show more distinct differences compared to phyllite. The 3620 cm^−1^ band is prominent, and its intensity is much higher than neighboring bands. Russell et al. (1970) interpreted the 3624 cm^−1^ montmorillonite band as derived from OH stretching vibrations in Al_2_OH and AlMgOH. Bending vibrations of these groups give bands at 915 cm^−1^ (Al_2_OH) and much weaker at 843 cm^−1^ (AlMgOH). After Eu adsorption, the intensity of the 915 cm^−1^ band increased slightly, possibly as a result of Eu binding to OH groups. Weakening of bands intensity after Eu adsorption occurred below 900 cm^−1^, i.e., in the region of OH deformation vibrations in aluminol and silanol groups (Vaculíková and Plevová 2005). The description of the Phy/B mixture spectra is presented in the Supporting Information.

### Adsorption kinetics

**Fig. 2:**
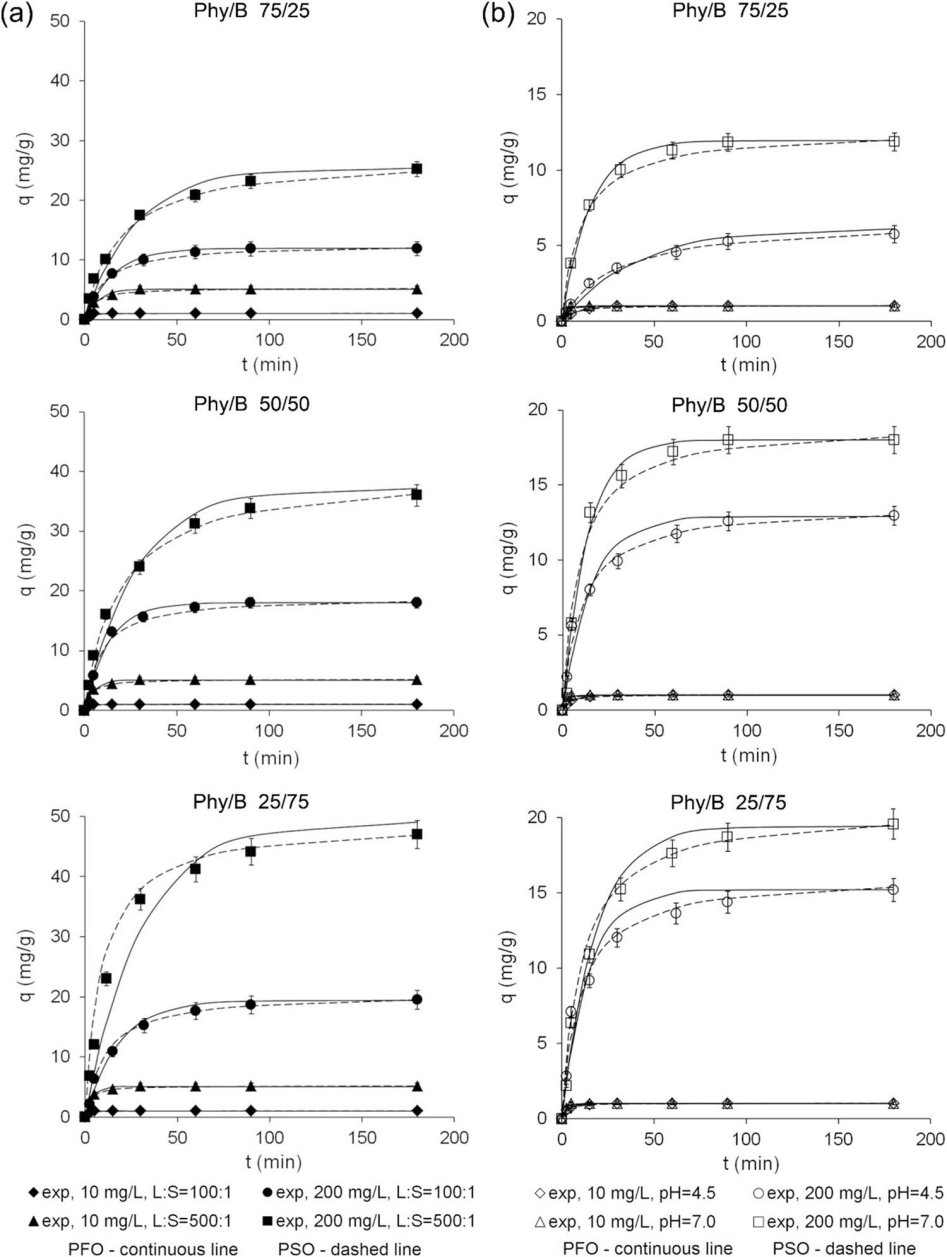
Isothermal (23 ± 2 °C) time-dependent Eu(III) adsorption onto Phy/B mixtures for different L/S ratio at solution pH 7.0 (**a**) and at different initial solution pH for L:S 100:1 (**b**); exp, experimental data

Adsorption kinetics was examined for 1400 min; however, the experimental results showed that 180 min was sufficient for reaching an equilibrium state in all Eu-adsorbent systems. There was a rapid and efficient one-step adsorption for *C*_0_ of 10 mg/L, whereas for *C*_0_ of 200 mg/L, a two-step reaction was observed with initial fast adsorption within 15 min followed by a slower equilibration. At the initial Eu concentration of 10 mg/L, adsorption onto each Phy/B mixture reached equilibrium within 10 min and at the initial Eu concentration of 200 mg/L — within 180 min (Fig. 2). The two-step process is attributed to a fast, diffusion-controlled surface reaction followed by a rate-limiting step that can be explained by a variety of processes including surface precipitation, diffusion into micropores, structural arrangement, or surface-mediated reduction.

For the PFO and PSO models, the curve fitting parameters (adsorption rate constants, *k*_1_, and *k*_2_) and the equilibrium adsorbed concentration, *q*, were calculated (Table 2). High correlation coefficients (*R*^2^ > 0.95) were obtained for the non-linear plots of the pseudo-first Lagergren and pseudo-second-order rate equations. Statistical analysis showed that the pseudo-second-order model resulted in better correlations compared to the pseudo-first-order model for *C*_0_ of 200 mg/L and all pH values and L/S ratios. The pseudo-first-order equation fitted well experimental data for *C*_0_ of 200 mg/L for the first 20–30 min of the adsorption process (Fig. 2). According to Ho and McKay (1998), the pseudo-first-order model applies only to a limited fraction of the reaction range. For this reason, the pseudo-second-order model was used to determine the rate constant, *k*_2_, and the Eu(III) equilibrium adsorbed concentration, *q*, with respect to *C*_0_, L/S, and pH. Estimated *q* was similar to or a little higher than experimentally determined adsorption capacity (Table 2) and depended on the B content in the Phy/B mixtures for *C*_0_ of 200 mg/L. At *C*_0_ of 10 mg/L, the *q* was constant regardless of the B content in the mixtures (Fig. 2a). A small difference in *k*_2_ determined for various Phy/B mixtures suggests that B content in the mixtures was less important than other experimental conditions. The *k*_2_ strongly depended on the initial Eu(III) concentration, pH, and L/S ratio. It was higher for L:S of 100:1 than for 500:1 for both initial Eu concentrations and all of the Phy/B mixtures. However, the effect of L/S was higher for *C*_0_ of 10 than 200 mg/L (Table 2). The increase in *k*_2_ with increasing content of adsorbents presumably was due to an elevated number of total adsorption sites, including strong adsorption sites in montmorillonite (Bradbury and Baeyens 2002, 2005; Bradbury et al. 2005).

**Table 2:** Kinetics models fitting parameters for adsorption of Eu(III) ions onto Phy/B mixtures compared to phyllite (Phy) and bentonite (B)

	Phy		Phy/B = 75/25		Phy/B = 50/50		Phy/B = 25/75		B	
	PFO	PSO	PFO	PSO	PFO	PSO	PFO	PSO	PFO	PSO
pH 4.5, *C*_*0*_ 10 mg/L, L:S 100:1										
*q*	1.011		1.011		1.011		1.011		1.012	
*q*_*e*_	1.019	1.069	1.009	1.032	1.008	1.047	1.008	1.047	1.01	1.042
*k*_*1*_, *k*_*2*_	0.2248	0.3403	0.2064	0.3338	0.2074	0.3416	0.2059	0.3457	0.2029	0.3515
*R*^*2*^	0.9516	0.9455	0.9985	0.9796	0.9954	0.9879	0.9964	0.9948	0.9899	0.9809
*SSE*	0.0772	0.1429	0.0080	0.0271	0.0153	0.0138	0.0033	0.0138	0.0093	0.0118
*χ*^*2*^	0.0291	0.0913	0.0011	0.0293	0.0161	0.0093	0.0031	0.0041	0.0111	0.0137
pH 4.5, *C*_*0*_ 200 mg/L, L:S 100:1										
*q*	3.50		6.60		13.65		16.80		19.71	
*q*_*e*_	3.189	3.510	6.1189	6.671	12.91	13.73	15.19	16.81	19.71	21.39
*k*_*1*_,* k*_*2*_	0.0532	0.00833	0.0455	0.0074	0.0456	0.0062	0.0416	0.0043	0.0352	0.0016
*R*^*2*^	0.9485	0.9891	0.9778	0.9975	0.9605	0.9913	0.9456	0.9858	0.9973	0.9869
*SSE*	0.7471	0.1586	1.435	0.1792	3.453	1.8812	10.02	5.260	1.823	5.580
*χ*^*2*^	0.3649	0.0926	0.4186	0.0451	1.098	0.2522	0.714	0.794	0.2663	0.3052
pH 7.0, *C*_*0*_ 10 mg/L, L:S 100:1										
*q*	1.01		1.014		1.014		1.014		1.01	
*q*_*e*_	1.008	1.042	1.018	1.033	1.019	1.039	1.019	1.034	1.014	1.038
*k*_*1*_,* k*_*2*_	0.4837	0.6926	0.4831	0.6865	0.4686	0.6060	0.4396	0.5832	0.3316	0.5801
*R*^*2*^	0.9889	0.9695	0.9829	0.9763	0.9909	0.9704	0.9912	0.9712	0.9891	0.9632
*SSE*	0.0124	0.0339	0.0173	0.0442	0.0089	0.0289	0.0086	0.0281	0.0115	0.0386
*χ*^*2*^	0.0085	0.0205	0.0116	0.0287	0.0065	0.0196	0.0063	0.0319	0.0071	0.0246
pH 7.0, *C*_*0*_ 200 mg/L, L:S 100:1										
*q*	7.20		12.0		18.07		19.57		20.6	
*q*_*e*_	7.053	7.741	11.96	12.71	18.03	19.11	19.42	20.60	20.06	21.52
*k*_*1*_,* k*_*2*_	0.0561	0.0058	0.0658	0.0055	0.0659	0.0057	0.0676	0.0051	0.0558	0.0039
*R*^*2*^	0.9949	0.9960	0.9909	0.9701	0.9914	0.9672	0.9923	0.9882	0.9926	0.9902
*SSE*	0.4169	0.3211	2.248	2.520	6.034	5.358	5.485	4.884	1.0814	4.5168
*χ*^*2*^	0.2075	0.0891	0.0713	0.2335	0.1288	0.4789	0.6092	0.2332	0.0661	0.2908
pH 7.0, *C*_*0*_ 10 mg/L, L:S 500:1										
*q*	4.32		5.013		5.013		5.013		5.13	
*q*_*e*_	4.358	4.891	5.084	5.526	5.083	5.275	5.085	5.233	4.416	5.039
*k*_*1*_,* k*_*2*_	0.2433	0.0561	0.2452	0.0553	0.2145	0.0538	0.2151	0.0517	0.2011	0.0485
*R*^*2*^	0.9929	0.9809	0.9921	0.9861	0.9914	0.9896	0.9923	0.9954	0.9934	0.9968
*SSE*	0.1184	0.1104	0.2493	0.4404	0.2440	0.2981	0.1998	0.1191	0.5279	0.0851
*χ*^*2*^	0.0284	0.1524	0.0658	0.0665	0.0561	0.0427	0.0404	0.0194	0.1181	0.0163
pH 7.0, *C*_*0*_ 200 mg/L, L:S 500:1										
*Q*	22.09		26.4		38.9		48.5		63.84	
*q*_*e*_	20.08	21.54	25.42	27.39	37.22	40.03	49.18	49.24	61.89	66.11
*k*_*1*_,* k*_*2*_	0.0473	0.00325	0.0333	0.0013	0.0362	0.00133	0.0364	0.0013	0.0366	0.00085
*R*^*2*^	0.9423	0.9879	0.9837	0.9931	0.9933	0.9949	0.9839	0.9541	0.9819	0.9936
*SSE*	147.6	5.241	9.159	2.906	7.545	1.231	27.25	71.39	42.63	12.67
*χ*^*2*^	11.73	0.4343	0.3529	0.2445	0.1939	0.1717	0.7941	2.299	0.9813	0.5506

*q*_*e*_ (mg/g) the Eu(III) adsorption at equilibrium, *k*_*1*_ (1/min) adsorption rate constant for PFO, *k*_*2*_ (g/mg·min) adsorption rate constant for PSO

The Eu(III) adsorption rate increased with increasing pH (Fig. 2b) at *C*_0_ of 10 mg/L. This observation can be explained by the mineral surface becoming increasingly negatively charged and, hence, attracting the Eu cations due to electrostatic interactions. There is no correlation between *k*_2_ and pH for *C*_0_ of 200 mg/L, apparently, because the electrostatic interactions had a small share in bonding Eu(III) at the high initial concentration.

The PSO rate constant strongly depended on the initial Eu(III) concentration (Fig. 2a, b). Adsorption rate constant increased with decreasing Eu(III) concentration because there was a larger fraction of binding sites in the adsorbent available for the Eu(III) ions. The higher the initial Eu(III) concentration, the longer it takes to reach equilibrium (Plazinski et al. 2009).

The observed effect of the initial Eu(III) concentration on *k*_2_ was approximately 2 orders of magnitude higher at a lower L/S (Table 2). According to Al-Degs et al. (2006), an inverse relationship between initial Eu(III) concentration and *k*_*2*_ in adsorption by a natural adsorbent is due to the effect of increased competition for the limited number of active adsorption sites by the adsorbate at higher initial concentrations of heavy metals. The observed decrease in *k*_2_ with increasing Eu(III) concentrations can be explained by the occurrence of strong and weak adsorption sites. At high concentrations, Eu(III) ions tend to preferentially occupy strong binding sites and then weaker ones in clay minerals of the Phy/B mixtures.

The pseudo-second-order kinetic model assumes that the adsorption rate is controlled by chemisorption (He et al 2020), whereas the adsorption capacity is controlled by the number of active adsorption sites (Robati 2013).

### Removal efficacy of Eu(III) by the Phy/B mixtures

**Fig. 3:**
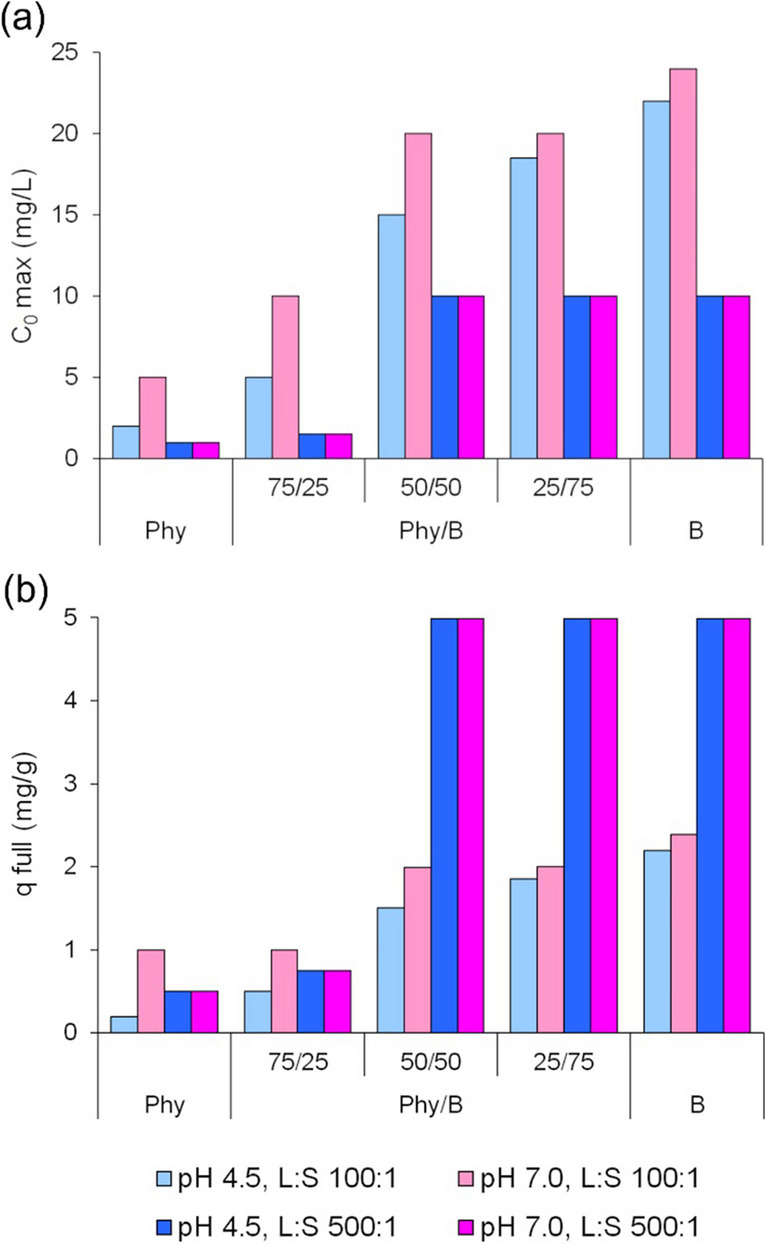
Maximum initial Eu(III) concentration in solution, *C*_0*max*_ (**a**) and full adsorption of Eu(III) ions by the Phy/B mixtures (**b**) for the total removal efficacy *RE* > 99.99% and *C*_*eq*_ < 0.0001 mg/L

The maximum initial concentration, *C*_0*max*_, depended on the B content in the Phy/B mixtures (Fig. 3a). The highest impact of the B content on *C*_0*max*_ was observed for the 50Phy/50B mixture. The highest *C*_0*max*_ and the most favorable adsorption conditions were at pH 7 and L:S 100:1. No effect of the initial solution pH on *C*_0*max*_ was observed at L:S 500:1. The full adsorption of the 50Phy/50B mixture was close to the full adsorption of B at L:S of 500:1 for both pH values, and it was 0.68 and 0.79 of B adsorption capacity at L:S of 100:1 and pH 4.5 and 7.0, respectively (Fig. 3b).

**Fig. 4:**
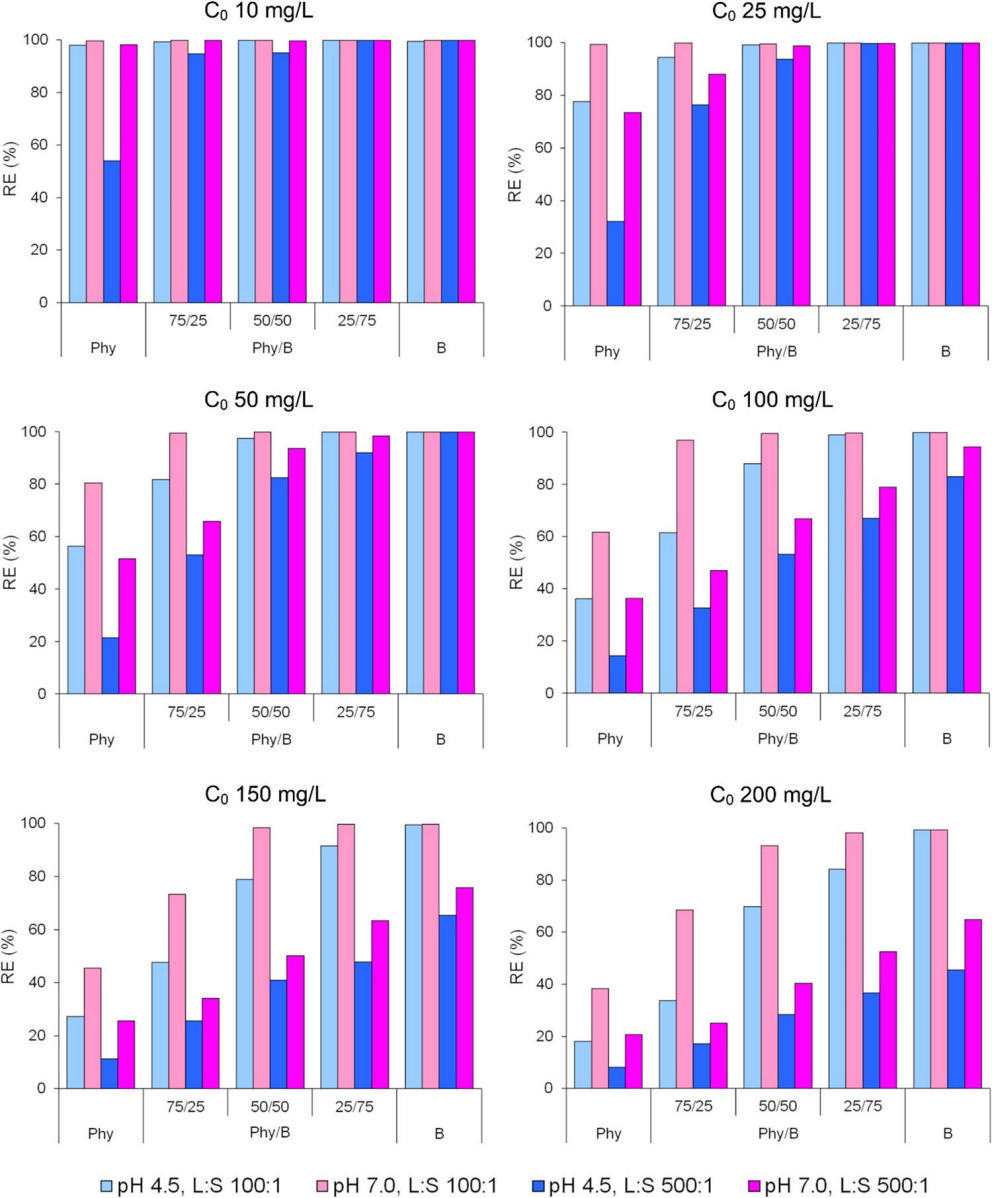
Removal efficacy of Eu(III) by the Phy/B mixtures as a function of initial Eu(III) concentrations

Changes in *RE* of Eu(III) with increasing Eu(III) initial concentrations ranging from 10 to 200 mg/L were investigated for four pH-L/S systems and six concentrations (Fig. 4). Removal efficacy decreased as the initial Eu concentration increased, and it depended not only on Phy/B but also on L/S and the initial solution pH.

For the Eu(III) initial concentrations of 10–50 mg/L at pH 7.0 and L/S 100:1, Eu(III) ions were removed from the solution by the adsorption on the Phy/B mixtures as efficiently as on B. A stronger impact of pH and L/S on *RE* was observed at lower contents of B in the mixtures, whereas a higher impact of L/S than pH was observed in all Phy/B mixtures. The effect of pH was usually higher at L/S 100:1 than at 500:1 (Fig. 4).

Multi-way analysis of variance was used to study the effect of pH, L/S ratio, proportions of Phy to B in the mixtures, and Eu(III) initial concentration on adsorption efficacy of the Phy/B mixtures (Supporting Information).

### Adsorption capacity of the Phy/B mixtures and mechanisms of Eu(III) bonding

**Fig. 5:**
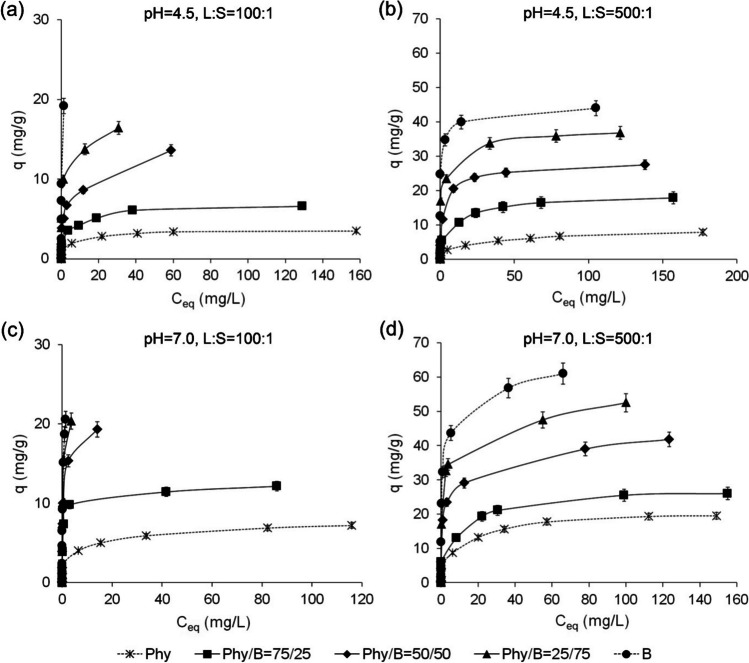
The adsorption capacity of the Phy/B mixtures for Eu(III) ions

The adsorption capacity of the Phy/B mixtures for Eu(III) increased with the increase in B content depending on the L/S and pH (Fig. 5). The adsorption system with the highest Eu(III) initial concentration did not reach the complete saturation for the Phy/B mixtures of 50/50 and 25/75 at L/S 100:1, regardless of pH as indicated by the lack of plateau on the adsorption curves (Fig. 5(a, b, c)). The adsorption capacities of the Phy/B mixtures for Eu(III) expressed in cmol_+_/kg ranged from 0.51 to 0.65 of their CECs. High adsorbent concentration at L:S of 100:1 and a high number of total adsorption sites resulted in the partial occupation of the adsorption centers at *C*_0_ of 200 mg/L because of the high cation exchange capacity of montmorillonite in B (Table 1). The 75Phy/25B mixtures adsorbed 6.6 mg/g of Eu(III) at pH 4.5 and 12.14 mg/g at pH 7.0, i.e., 0.60 and 1.1 of CEC, respectively. High concentrations of the Phy/B mixture in solution enhance the possibility of collision between the suspended solid particles of the adsorbents leading to their aggregation. The aggregated particles have lower total surface area which results in a decrease in their adsorption capacity (Yu et al. 2015).

With an increase in the L/S to 500:1, i.e., a decrease in the adsorbent concentration, the adsorption capacity of the Phy/B mixtures for Eu(III) ions increased (Fig. 5(c, d)).

The maximum adsorption capacity of all Phy/B mixtures was higher than their CEC and increased 1.19–1.62-fold at the solution initial pH 4.5 and 1.7–2.4-fold at pH 7.0. Therefore, a mechanism other than ion exchange must have been responsible for the Eu-binding.

**Fig. 6:**
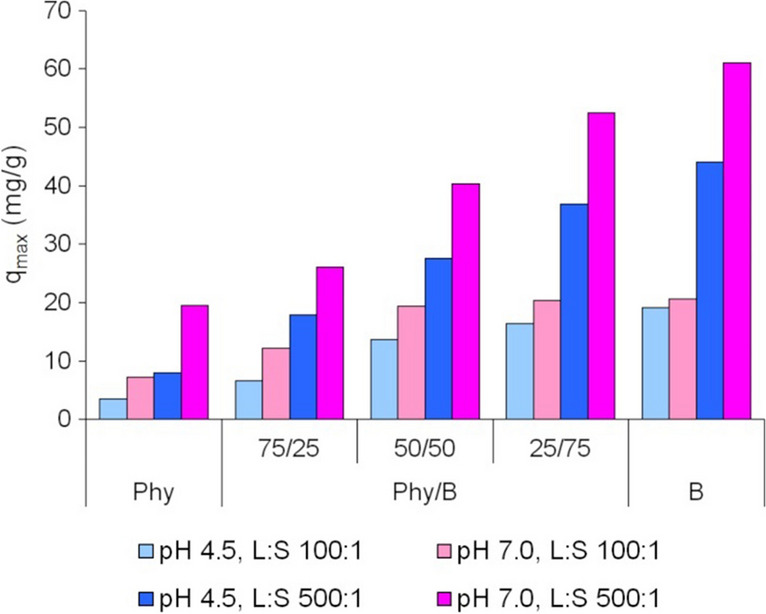
The maximum adsorption capacity of phyllite, bentonite, and their mixtures

The adsorption capacity of Phy and Phy/B mixtures was higher at the solution initial pH of 7.0 than 4.5, probably due to the precipitation of Eu(OH)_3_ at pH 7.0, both on the surface of Phy/B mixtures and Phy (Fig. 1). The comparison of the maximum adsorption capacity of different Phy/B mixtures reveals the highest increase in adsorption capacity for 75Phy/25B relative to Phy (1.33–2.26-fold increase) and for 50Phy/50B relative to 75Phy/25B (1.53–2.0-fold increase) (Fig. 6).

Speciation of Eu(III), surface properties of the mixtures constituents, and protonation/deprotonation reactions of amphoteric surface functional groups (Si–OH, Al_2_–OH) in clay minerals were all affected by solution pH.

**Fig. 7:**
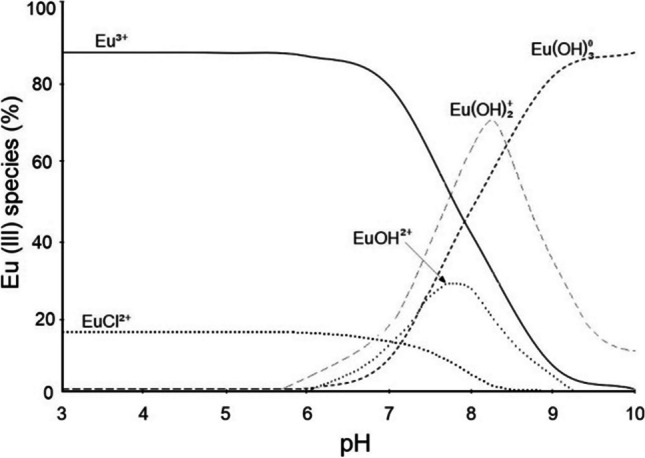
Distribution of hydrolysed products of Eu(III) at 25 °C in aqueous solution

Europium may occur in solution at different valencies: Eu^3+^ and EuCl^2+^ at pH < 5 and Eu^2+^ in hydrolyzed species, i.e., Eu(OH)^2+^ and Eu(OH)_2_^+^ at pH > 5.5 (Fig. 7).

According to Bradbury and Baeyens (2002), hydrolysis of Eu does not occur until pH ~ 6 because of the large Eu ionic radius (0.95 Å). The presence of Eu(OH)^2+^ and Eu(OH)_2_^+^ ions in solution suggests that they can be bound by hydrogen bonds to the Si–OH and/or Al_2_–OH surface functional groups of clay minerals in the Phy/B mixtures according to the reactions:3

4The hydrolyzed species are more hydrophobic than trivalent cations. From the Fig. 7, it can be inferred that Eu(OH)_3_ precipitates at pH > 6.0. However, high adsorbent concentration in the solution with the L:S ratio of 100:1 and high initial concentration of Eu(III) (100–200 mg/L) enables precipitation of Eu(OH)_3_ even at pH ≤ 7.0 as it was the case in this study (Fig. 1).

**Fig. 8:**
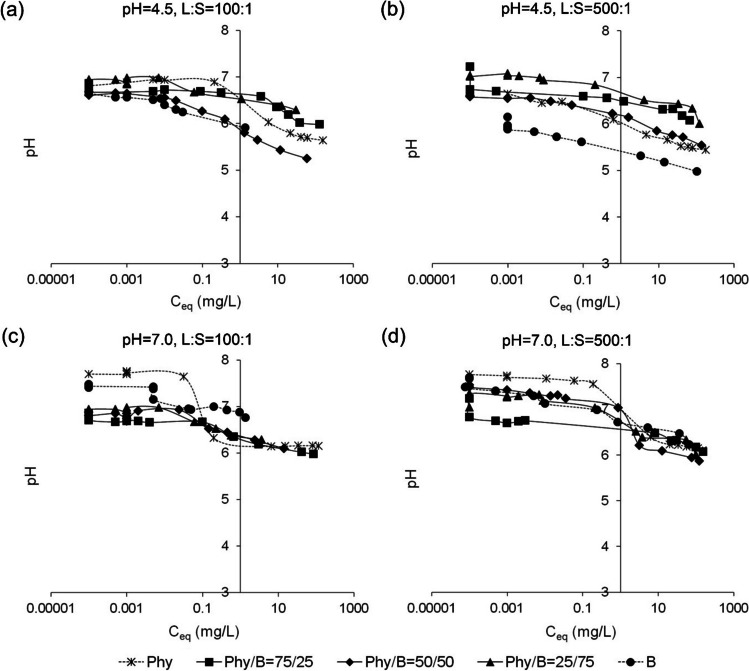
Changes of pH in equilibrium solutions

The equilibrium pH of the Ph/B mixtures suspension decreased as the concentration of Eu(III) in the initial solution increased (Fig. 8) and ranged between 6.89 and 5.25 for L/S 100:1 at initial pH 4.5 and 7.0 and L/S 500:1 at pH 4.5. The equilibrium pH ranged from 7.53 to 5.87 at L/S 500:1 and pH 7.0 (Fig. 8). Equilibrium pH higher than the initial one results in good buffer properties of bentonite and removal of Ca^2+^ and Mg^2+^ ions from montmorillonite (Aitken et al. 1990).

The surface functional groups of minerals are protonated/deprotonated depending on the pH, according to the following equations:

5 $$\begin{array}{ccc}-{\text{Si}}-\text{OH+ }{\text{H}}^{+}\leftrightarrow -{\text{Si}}-{\text{O}}{\text{H}}_{2}^{+}& \mathrm{or}& \begin{array}{cc}-{\text{Al}}_{2}-\text{OH} + {\text{H}}^{+}\leftrightarrow -{\text{Al}}_{2}-{\text{O}}{\text{H}}_{2}^{+}& \mathrm{pH}<{\mathrm{pH}}_{\mathrm{PZC}}\end{array}\end{array}$$

6 $$\begin{array}{ccc}-\mathrm{Si}-\mathrm{OH}\leftrightarrow \mathrm{Si}-{\mathrm{O}}^{-}+{\mathrm{H}}^{+}& \mathrm{or}& \begin{array}{cc}-{\text{Al}}_{2}-{\text{OH}}\leftrightarrow -{\text{Al}}_{2}-{\text{O}}^{-}\text{+}{\text{H}}^{+}& \mathrm{pH}>{\mathrm{pH}}_{\mathrm{PZC}}\end{array}\end{array}$$

At the initial Eu(III) concentrations between 0.01 and 35 mg/L, the equilibrium solution pH > pH_PZC_ (Table 1), resulting in a negative surface charge of the mixtures-constituting minerals. Europium ions can be electrostatically bound to the negatively charged mineral surfaces according to the reaction:

7 $$-\mathrm{Si}-{\mathrm{O}}^{-} + {\mathrm{Eu}}^{3+}\to -\mathrm{Si}-{\mathrm{O}}^{-} \cdot \cdot \cdot \cdot \cdot \cdot \cdot \cdot \cdot \cdot {\mathrm{Eu}}^{3+}$$

8 $$-{\mathrm{Al}}_{2}-{\mathrm{O}}^{-}+ {\mathrm{Eu}}^{3+} \to -{\mathrm{Al}}_{2}-{\mathrm{O}}^{-}\cdot \cdot \cdot \cdot \cdot \cdot \cdot \cdot \cdot \cdot {\mathrm{Eu}}^{3+}$$

At *C*_0_ > 35 mg/L, the surface charge of the Phy/B mixtures was positive due to the protonation reaction of the surface hydroxyl groups; hence, repulsive forces occur between Eu(III) and mineral surfaces.

As a consequence of the layered structure of clay minerals in the Phy/B mixture, Eu(III) ions are adsorbed by:

1. Forming the outer-sphere complexes via cation exchange within the interlayer space in montmorillonite (Liu et al. 2023) and by the attachment to the permanently negatively charged sites on external basal surfaces of montmorillonite at low pH:

9 $$3{X}^{2-}-3{\mathrm{Ca}}^{2+}+ 2{\mathrm{Eu}}^{3+}+4\left({\mathrm{H}}_{2}\mathrm{O}\right)\leftrightarrow 3{\mathrm{X}}^{2-}-2\mathrm{Eu}({\mathrm{H}}_{2}\mathrm{O}{)}_{2}^{3+}+3{\mathrm{Ca}}^{2+}$$

2. The formation of the Eu hydroxyl surface inner-sphere complex composed of the cation and silanol (Si–OH) or aluminol (Al_2_–OH) groups at the crystal edges in both bentonite and phyllite:

10 $$\begin{array}{ccc}-\mathrm{Si}-{\mathrm{O}}^{-}+ {\mathrm{Eu}}^{3+} \leftrightarrow -\mathrm{Si}-{\mathrm{OEu}}^{2+}& or& -{\mathrm{Al}}_{2}-{\mathrm{O}}^{-}+ {\mathrm{Eu}}^{3+}\leftrightarrow -{\mathrm{Al}}_{2}-{\mathrm{OEu}}^{2+}\end{array}$$

The inner-sphere complexation at the edges occurs with increased pH (Geckeis et al. 2013).

According to the two-site protolysis non-electrostatic surface complexation and cation exchange (2SPNE SC/CE) model of Bradbury and Baeyens (2002), the Ln/An uptake by montmorillonite can be explained by (i) cation exchange on planar sites, (ii) surface complexation on strong sites (≡S^S^OH) with high affinity, but low capacity, and (iii) surface complexation on weak sites (≡S^W1,2^OH) with high capacity and low affinity. According to Gao et al. (2021), Am(III) adsorption on Gaomiaozi bentonite may be described by the surface complexation model including cation exchange species ((≡X)_3_Am) and three surface complexes (≡S^S^OAm^2+^, ≡S^S^OAm(OH)^+^, and ≡S^S^OAm(OH)_2_). Fernandes et al. (2016) observed that Ln/An^3+^ strong inner-sphere complexes were formed at low loadings through binding to three Al(O,OH)_6_ octahedra, most likely by occupying vacant sites in the octahedral layers of montmorillonite, which are exposed on {010} and {110} edge faces. At higher loadings, Ln/An^3+^ were bonded only via one Al octahedron, forming a weaker, edge-sharing surface complex.

Iron oxyhydroxides in phyllite are additional active centers for the binding of Eu(III).

This study revealed the dependence of the edge site adsorption capacity of the Phy/B mixtures on the surface area. The 25Phy/75B mixture had the highest specific surface area and the highest adsorption capacity. Moreover, adsorption capacity increased with increasing CEC of the Phy/B mixtures. The contribution of individual mechanisms to the binding of Eu(III) by the Phy/B mixtures depends on the amount of bentonite.

### Adsorption isotherms

**Fig. 9:**
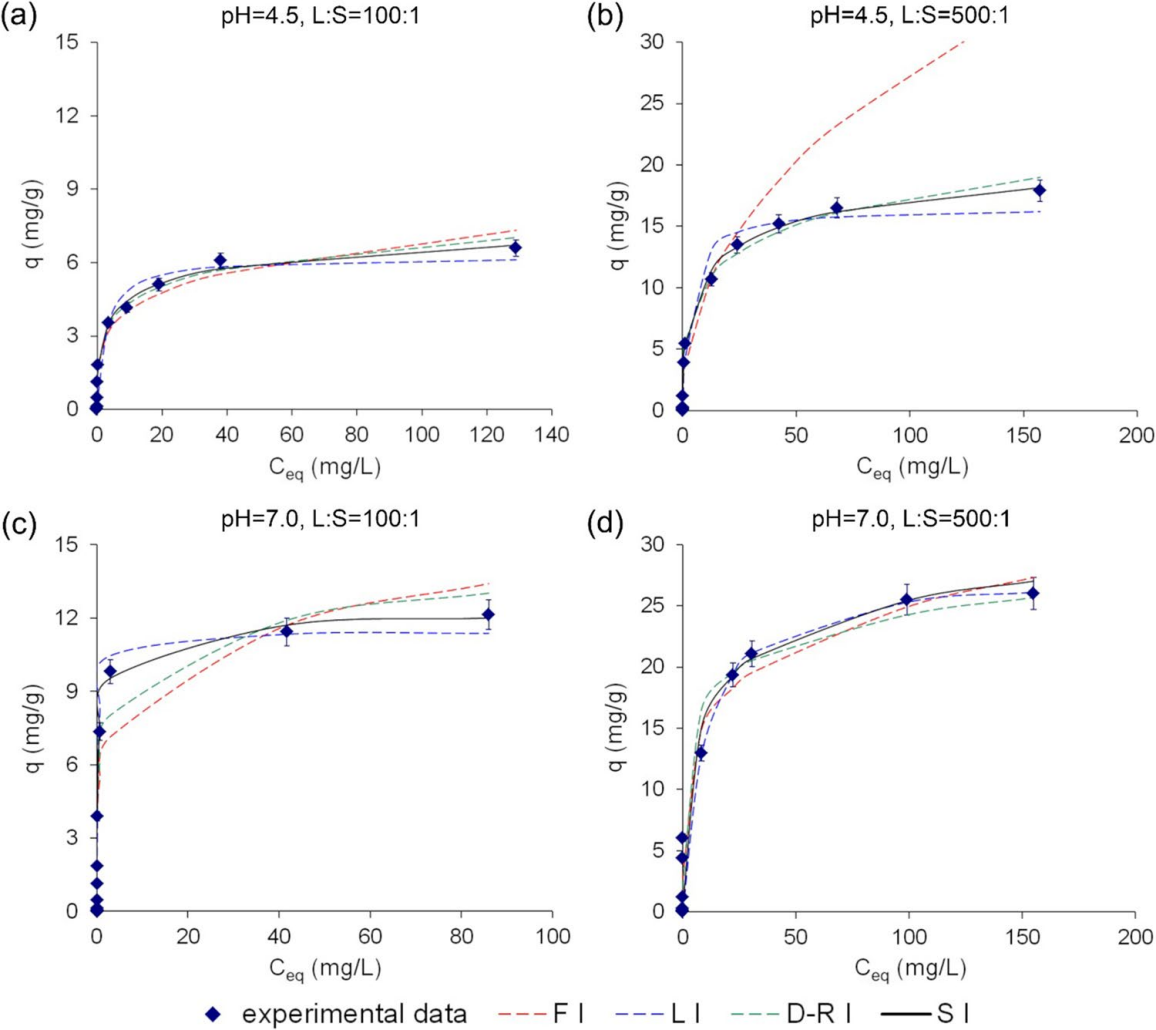
Experimental data and predicted adsorption isotherms of Eu(III) for the Phy/B mixture of 75/25

The parameters of isotherm models determined by the non-linear regression analysis, values of determination coefficient, and non-linear error functions are summarized in Table 3, and the fits of experimental data with four models are shown in Fig. 9.

**Table 3:** The isotherm constants for the adsorption of Eu(III) ions onto Phy/B mixtures

	Phy/B					Phy/B				
	100/0	75/25	50/50	25/75	0/100	100/0	75/25	50/50	25/75	0/100
	pH 4.5, L:S 100:1					pH 7.0, L:S 100:1				
Freundlich isotherm										
*1/n*	0.2127	0.2306	0.2431	0.2705	0.3133	0.2282	0.2476	0.2571	0.2885	0.4141
*K*_*F*_	1.341	2.389	4.374	7.994	17.97	2.541	5.813	10.61	15.07	17.34
*R*^*2*^	0.9711	0.9737	0.9877	0.9654	0.9076	0.9848	0.9161	0.9612	0.9608	0.9891
*SSE*	0.37	1.86	2.03	6.03	19.61	1.38	20.44	12.30	10.49	7.35
*χ*^*2*^	0.29	1.60	1.78	3.95	7.72	1.85	7.58	3.07	7.01	3.41
Langmuir isotherm										
*q*	3.50	6.60	13.62	16.42	19.16	7.20	12.14	19.33	20.37	20.56
*q*_*L*_	3.512	6.649	13.68	16.48	19.80	7.24	12.41	19.38	20.46	21.22
*K*_*L*_	0.2537	0.3684	0.5203	9.661	27.38	2.821	3.658	3.784	6.315	7.291
*R*^*2*^	0.9499	0.9635	0.9373	0.9474	0.9884	0.9297	0.9747	0.9674	0.9607	0.9729
*SSE*	0.77	1.10	1.43	1.49	1.95	6.44	2.49	6.26	10.56	8.35
*χ*^*2*^	2.71	0.75	7.96	1.27	0.64	2.79	2.14	2.94	3.42	5.10
Dubinin-Raduskevich isotherm										
*q*_*D*_	0.0400	0.0810	0.1580	0.2010	0.5660	0.0810	0.1331	0.3210	0.4650	0.9980
*β·10*^*−3*^	1.677	1.849	1.923	1.409	1.796	1.724	1.299	1.672	1.74	1.807
*E*	17.26	16.44	16.12	18.84	16.68	17.03	19.62	17.29	16.95	16.63
*R*^*2*^	0.9890	0.9929	0.9767	0.9838	0.9276	0.9932	0.9633	0.9856	0.9806	0.9909
*SSE*	0.22	0.51	2.21	3.19	2.87	0.61	2.33	4.90	5.17	3.15
*χ*^*2*^	0.40	0.46	0.98	2.35	3.73	1.03	2.38	2.88	2.32	2.31
Sips isotherm										
*q*_*S*_	4.892	8.883	16.67	20.09	31.12	12.67	12.67	23.69	27.23	59.39
*K*_*S*_	0.4069	0.3816	0.5053	0.9750	1.451	0.2721	1.007	1.168	1.563	0.4178
*m*_*S*_	0.3869	0.4318	0.3909	0.5867	0.3614	0.3293	0.5262	0.5037	0.5187	0.4996
*R*^*2*^	0.9909	0.9961	0.9935	0.9853	0.9697	0.9919	0.9946	0.9962	0.9893	0.9902
*SSE*	0.19	0.27	0.82	1.56	8.30	0.73	0.77	1.83	5.60	4.66
*χ*^*2*^	0.18	0.28	0.92	1.59	4.32	1.19	1.13	2.30	2.91	2.66
	pH 4.5, L:S 500:1					pH 7.0, L:S 500:1				
Freundlich isotherm										
*1/n*	0.1964	0.2567	0.3281	0.1991	0.1316	0.1994	0.2059	0.2274	0.2114	0.2183
*K*_*F*_	0.4725	5.3825	8.977	15.37	25.47	0.5155	9.671	14.776	21.07	26.13
*R*^*2*^	0.9299	0.9807	0.9807	0.9664	0.9013	0.9441	0.9739	0.9659	0.9645	0.9219
*SSE*	0.0974	6.46	11.72	12.49	293.6	0.1014	5.38	7.56	12.24	156.3
*χ*^*2*^	0.163	5.31	3.11	4.02	1.63	0.1823	6.15	5.40	3.65	1.93
Langmuir isotherm										
*q*	7.90	17.9	27.5	36.8	44.00	19.5	26.00	41.80	52.50	61.0
*q*_*L*_	7.973	17.95	27.71	37.15	40.47	20.56	27.70	42.33	53.26	61.45
*K*_*L*_	0.084	0.2958	0.4557	0.537	21.18	0.1089	0.1060	0.6917	1.129	1.587
*R*_*2*_	0.9366	0.9711	0.9870	0.9624	0.9815	0.9488	0.9611	0.9617	0.9629	0.9482
*SSE*	4.74	1.09	2.19	2.70	31.96	2.347	1.88	2.65	2.88	143.8
*χ*^*2*^	0.304	1.64	2.67	3.71	1.24	0.0126	1.23	2.80	2.87	1.559
Dubinin-Raduskevich isotherm										
*q*_*D*_	0.1030	0.237	0.351	0.391	0.4110	0.2470	0.258	0.481	0.586	0.7090
*β·10*^*−3*^	1.361	1.934	1.923	1.408	1.231	2.11	1.472	1.76	1.541	1.562
*E*	14.05	16.07	16.12	18.44	20.18	15.31	18.43	16.83	18.03	17.92
*R*^*2*^	0.9909	0.9935	0.9767	0.9829	0.9879	0.9844	0.9705	0.9891	0.9851	0.9820
*SSE*	0.676	1.67	2.14	4.43	13.26	0.545	1.54	2.70	3.53	34.06
*χ*^*2*^	0.054	0.872	1.23	2.44	0.634	0.231	1.11	1.13	1.79	0.543
Sips isotherm										
*q*_*S*_	10.76	23.68	30.39	41.25	45.21	25.79	37.15	49.21	58.14	77.56
*K*_*S*_	0.0631	0.2603	0.4561	0.8075	18.99	0.1961	0.2877	0.5274	0.9594	0.6555
*m*_*S*_	0.3307	0.5017	0.6408	0.4226	0.8174	0.3785	0.4612	0.4613	0.4722	0.4047
*R*^*2*^	0.9928	0.9973	0.9931	0.9881	0.9811	0.9944	0.9748	0.9947	0.9967	0.9865
*SSE*	1.87	0.68	1.48	1.09	1.957	0.466	1.51	1.88	4.16	0.4614
*χ*^*2*^	0.154	0.452	0.841	0.772	0.233	0.152	0.57	1.23	1.81	0.0629

*K*_*F*_ ((mg/g)·(L/mg)^1/*n*^); *q*_*L*_ (mg/g); K_L_ (L/mg); *q*_*D*_ (mmol/g); *β* (mol^2^/J^2^); *E* (kJ/mol); *q*_*S*_ (mg/g); *K*_*S*_ (L/mg)^*m*^

The isotherm equations described the Eu(III) adsorption on the Phy/B mixtures very well, as suggested by the regression coefficients (*R*^2^) ranging from 0.9161 to 0.9877 for the Freundlich model and from 0.9748 to 0.9962 for the Sips model (Table 3). Values of 1/*n* estimated from the Freundlich isotherm (Eq. (S1) — Table S1) were below 1 (0.1991–0.3281) and did not depend on experimental conditions. They were indicative of chemical adsorption of Eu(III) ions on all mixtures. The good adsorption capacity of the Phy/B mixtures was indicated by *n* > 2. Values of *K*_*F*_ were higher for mixtures with a high content of bentonite and indicated a higher adsorption intensity at pH 7.0 and L/S 500:1 than at pH 4.5 and L/S 100:1. The fitting of adsorption data to the Freundlich isotherm assumes surface heterogeneity and confirms the high porosity of the Phy/B mixture suggested by the heterogeneous adsorption not limited to the monolayer adsorption (Sharma and Tomar 2011).

Results of the error analysis showed that the Langmuir model better simulated the adsorption isotherms than the Freundlich model (Table 3) (He et al 2020). The Langmuir constant, *K*_*L*_ (Eq. (S2) — Table S1) varied depending on the adsorbent surface chemical composition and experimental conditions and was higher for the Phy/B mixture of 25/75 than 75/25. In addition, the effect of the experimental conditions on the affinity of the Phy/B mixture surface for Eu(III) and the bonding energy was similar to that of the *K*_*F*_ values, i.e., it increased with the increase in pH and L:S. The values of the maximum adsorption capacity (*q*_*L*_) were close to or higher than the theoretical (*q*_*max*_) values for all Eu(III)–Phy/B mixture systems. This observation suggests a monolayer Eu(III) adsorption model associated with ion exchange, in accord with previously obtained results for minerals used for Eu(III) removal from solutions (Hu et al 2010; Kautenburger and Beck 2010; Songsheng et al 2012).

Mean free energy values, *E* (Eq. (S4)—Table S1), for the Dubinin-Radushkevich isotherm was above 16 kJ/mol ranging from 16.07 to 19.62 kJ/mol as a result of a strong covalent interaction between Eu(III) and the silanol –Si–OH or aluminol –Al–OH groups on bentonite and phyllite surfaces.

The Sips model is a combination of two other models and represents systems in which one adsorbed molecule can occupy more than one adsorption site (Eq. (S5) — Table S1). The Sips model improved the fit of experimental data in the zone of higher curvature of the adsorption isotherms (Fig. 9). Using a third parameter, it logically improves the quality of the mathematical fit. The maximum adsorption capacity values (*q*_*S*_) for Eu(III) bonding obtained from the Sips model were higher than those derived from the Langmuir isotherm and than the experimentally determined. The parameter *K*_*S*_ changed in the same manner as the constants *K*_*L*_ of the Langmuir model. Differences between experimental and calculated values of adsorption capacity using the Langmuir and Freundlich models showed that at the lower initial concentration, the Freundlich model was a better choice for the characterization of adsorption but at higher initial concentrations; adsorption followed the Langmuir model.

The initial slope of the adsorption isotherm curve provides important information. A curve with a steep initial slope indicates that the adsorbent has a high affinity for Eu(III), and experimental conditions are most favorable. This affinity is indicated by the affinity constant derived from the Sips model; the highest affinity constant (*K*_*S*_ = 1.563) was obtained for the adsorption of Eu(III) on the 25Phy/75B mixture for the L:S 100:1 and pH 7.0.

### Distribution coefficient values for Eu(III) adsorbed on Phy/B mixtures

The laboratory batch sorption equilibrium test allowed the estimation of Eu(III) distribution coefficients (*K*_*d*_). This is an important parameter in transport modelling and one of the key parameters used in the safety analysis of spent fuel repositories (Lehto et al 2019). Distribution coefficients are relevant to the specific geochemical conditions of each system under study and give an indication of the extent of Eu(III) partitioning to the solid phase in different systems and are useful in assessing the migration of Eu(III) in the barrier. It can be defined as the ratio of the solid-phase concentration (*q*) to the aqueous phase concentration at equilibrium and can be expressed as:

11 $${K}_{d}=\frac{{C}_{0}-{C}_{eq}}{{C}_{eq}}.\frac{V}{m}=\frac{q}{{C}_{eq}}$$

If the adsorption is described by a non-linear isotherm, the partition coefficient is given by the equation:

12 $${K}_{d}=\frac{dq}{d{C}_{eq}}$$

and for the Sips isotherm, which best describes the adsorption of Eu(III) ions by the equation:

13 $${K}_{d}=\frac{\mathrm{q}{K}_{s}{mC}_{eq}^{m-1}}{({1+{K}_{s}{C}_{eq}^{m})}^{2}}$$

**Fig. 10:**
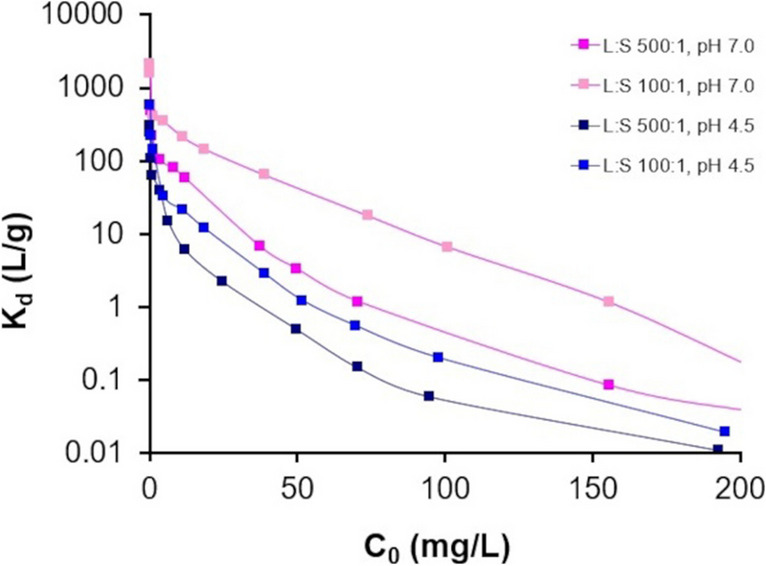
Distribution coefficient of Eu adsorption on 50Phy/50B mixture

The distribution coefficient was calculated for the Phy50/B50 mixture for the following conditions: initial concentration of Eu(III) 0.01–200 mg/L, L/S 100:1 and 500:1, initial concentration of Eu(III) 0.01–200 mg/L, pH 4.5 and 7.0, and is presented in Table 4, and the plots of the logK_d_ values for the adorption of Eu(III) on Phy/B of 50/50 against the initial concentration are shown in Fig. 10.

**Table 4:** Eu(III) distribution coefficient values (L/g)

Solid phase	L:S 500:1, pH 7.0	L:S 100:1, pH 7.0	L:S 500:1, pH 4.5	L:S 100:1, pH 4.5
Phy	526.1–0.00219	632.8–0.0188	64.22–0.0040	213.3–0.0023
Phy/B 75/25	867.4–0.0039	833.9–0.0427	242.2–0.0110	270.4–0.0055
Phy/B 50/50	1684–0.0261	2117–0.1276	302.88–0.0180	578.1–0.0443
Phy/B 25/75	2811–0.0356	3510–0.7159	1035–0.0153	2175–0.0726
B	3651–0.0694	5633–4.74	1432–0.0836	2929–1.903

The distribution coefficient values for Eu(III) are strongly dependent on the initial Eu(II) concentration, the pH of the solution, the L/S ratio, and the mineral composition of the solid phase.

The results show that the distribution coefficient values was:

- Higher for bentonite than for phyllite under all conditions and increased with the percentage of bentonite in the mixtures
- Higher for L/S of 100:1 than 500:1 for any initial concentration of Eu(III) ions
- Higher for pH 7.0 than 4.5
- Decreased with increasing initial Eu concentration

The lowest *K*_*d*_ value was observed for Eu(III) adsorption on Phy, and it increased with increasing bentonite in the mixture. Furthermore, as shown in Fig. 10, there were greater differences in *K*_*d*_ values for L/S 500:1 and 100:1 at pH 7.0 than at pH 4.5, and at pH 4.5 and 7.0 for L/S 100:1 than for 500:1. The results for Eu(III) adsorbed on bentonite at L:S 500:1, pH 4.5, and an initial concentration of 40 mg/L indicated that the *K*_*d*_ value was twice as high as the *K*_*d*_ value obtained by Pshinko et al. (2004) for Eu adsorption on Cherkasky montmorillonite under similar conditions.

## Conclusions

1. Batch experiments showed a rapid and efficient Eu(III) adsorption on all of the Phy/B mixtures investigated. The adsorption rate constant was strongly dependent on the initial Eu(III) concentration, pH, and L/S ratio. A high correlation of pseudo-second order kinetic model with experimental data showed that the adsorption rate was controlled by chemisorption, while the adsorption capacity was controlled by the number of active adsorption sites.
2. The adsorption capacity of the Phy/B mixtures for Eu(III) increased with the increase of the bentonite content in the Phy/B mixtures, but was dependent on both L/S and solution pH. The addition of bentonite to the mixtures had a strong effect on their CEC and specific surface area.
3. The highest increase in adsorption capacity of Phy/B mixtures compared to phyllite was observed for phyllite/bentonite ratios of 25/75 and 50/50. A greater effect of L/S than of solution pH on both the adsorption capacity and removal efficacy of Eu(III) was observed.
4. Europium can be adsorbed by (i) the outer-sphere complexes via cation exchange in the interlayer space of montmorillonite; (ii) attachment to the permanently negatively charged sites on the outer basal surfaces of montmorillonite; (iii) the inner-sphere complex of the hydroxyl surface composed of the cation and silanol (Si–OH) or aluminol (Al_2_–OH) groups at the crystal edges in both bentonite and phyllite; (iv) hydrogen bonding and attraction to a negatively charged mineral surface; and (v) precipitation as Eu(OH)_3_.
5. The Sips model showed the best fit to the experimental data, particularly in the zone of higher curvature on the adsorption isotherms indicating that adsorbed Eu(III) can occupy more than one adsorption site.
6. The results of this study show the advantage of the Phy/B mixtures in immobilizing actinides and certain fission products by combining the adsorption properties of montmorillonite and chlorite.

### Supplementary Information

Below is the link to the electronic supplementary material.

Supplementary file1 (DOCX 2.16 MB)

## Data Availability

The authors confirm that the data supporting the findings of this study are available within the article and its supplementary materials.
