# Supplementary material for: Phyllite/bentonite mixture—an alternative effective buffer material for a geological disposal of radioactive waste
Source: Environ Sci Pollut Res Int. 2023 Dec 8;31(2):2419–36. doi: 10.1007/s11356-023-31102-6 (PMC10791840; doi:10.1007/s11356-023-31102-6)
Supplement: Supplementary file 1 — Supplementary file1 (DOCX 2.16 MB) [file 11356_2023_31102_MOESM1_ESM.docx]

Supplement

Supporting Information for

Phyllite/bentonite mixture as a novel effective buffer material for a geological disposal of radioactive waste

Joanna Kyzioł-Komosińska^1)^, Janusz Janeczek^2)^, Agnieszka Dzieniszewska^1)^, Monika J. Fabiańska^2)^, Aniela Matuszewska^2)^, Ewa Teper^2)^, Ewa Szram^2)^, Tomasz Krzykawski^2)^, Magdalena Pająk^1)^

^1)^ Institute of Environmental Engineering Polish Academy of Sciences, 34 M. Skłodowska-Curie St., 41-819 Zabrze, Poland;

^2)^ Faculty of Natural Sciences, University of Silesia, 60 Będzińska St., 41-200 Sosnowiec, Poland

**Table S1** Isotherms equations

| Isotherm model | Equation | Parameters | Eq. |
| --- | --- | --- | --- |
| Freundlich | $q=K_{F}\cdot{C_{eq}}^{1/n}$ | *K_F_* – Freundlich isotherm constant ((mg/g)·(L/mg)^1/n^) and it is an approximate indicator of adsorption capacity  *1/n* – a function of the strength of adsorption in the adsorption process | S1 |
| Langmuir | $q=\frac{q_{L}K_{L}C_{eq}}{1+K_{L}C_{eq}}$ | *q_L_* – maximum monolayer coverage capacity (mg/g)  *K_L_* – Langmuir isotherm constant related to the affinity of binding sited (L/mg) | S2 |
| Dubinin-Radushkevich | $q=q_{D}\cdot exp\left( -\beta\varepsilon^{2} \right)$ | *q_D_* – the adsorption capacity of studied minerals for Eu(III) (mol/g)  *β* – the constant (mol^2^/J^2^)  *ε* – the Polanyi potential (J/mol) which is equal to  $\varepsilon=\text{RTln}(1+\frac{1}{C_{\text{eq}}})$.  *R* – the gas constant (8.314 J/mol K)  *T* – absolute temperature (K)  $E=\frac{1}{(2\beta)^{1/2}}$ – free energy (E) of the adsorption (kJ/mol) | S3  S4 |
| Sips | $q=\frac{q_{S}K_{S}{C_{eq}}^{m_{S}}}{1+K_{S}{C_{eq}}^{m_{S}}}$ | *q_S_* – Sips maximum adsorption capacity (mg/g)  *K_S_* – the Sips equilibrium constant (L/mg)^m^  *m_S_* – the Sips model exponent | S5 |

**Table S2** List of error function

| Error function | Abbreviation | Expression | Eq. |
| --- | --- | --- | --- |
| Sum of squares error | *SSE* | $\sum_{i=1}^{n} (q_{calc}-q_{meas})_{i}^{2}$ | S6 |
| Nonlinear chi-square test | *χ^2^* | $\sum_{i=1}^{n} \frac{(q_{meas}-q_{calc})^{2}}{q_{calc}}$ | S7 |

‘‘meas’’ and ‘‘calc’’ show the measured and calculated values; n is the number of observations in the experimental data

**Table S3** Kinetics equations

| Name of equation | Equation | Non-linear form | Description of kinetic parameters | Eq. |
| --- | --- | --- | --- | --- |
| Pseudo-first order (PFO) | $\frac{dq_{t}}{dt}=k_{1}(q_{e}-q_{t})$ | $q_{t}=q_{e}(1-e^{-k_{1}t})$ | *q_t_* (mg/g) - the Eu(III) adsorption at any given time t (min)  *q_e_* (mg/g) - the Eu(III) adsorption at equilibrium  *k_1_* (1/min) - adsorption rate constant for PFO  *k_2_* (g/mg min) - adsorption rate constant for PSO | S8 |
| Pseudo-second order (PSO) | $\frac{dq_{t}}{dt}=k_{2}(q_{e}-q_{t})^{2}$ | $q_{t}=\frac{k_{2}q_{e}^{2}t}{1+k_{2}q_{e}t}$ |  | S9 |

**Methods**

Scanning Electron Microscopy (SEM)

Scanning electron microscopy (SEM) was performed using analytical SEM Thermo Scientific Quanta 250 coupled with UltraDry EDS detector under the following operating conditions: accelerating voltage 15 kV; working distance ca. 10 mm; counting time 40 s. The spectral resolution for this detector is 130 eV for MnKα peak. The diameter of interaction volume was about 2 μm for aluminosilicates investigated, i.e., biotite, chlorite, and montmorillonite. EDS spectra of X-ray mapped grains with acquisition time 60 s were acquired before mapping to confirm the presence of analyzed elements. Europium was determined semi-quantitatively. Europium pentaphosphate EuP_5_O_14_ from the standards library of SPI Supplies was used as a standard for optimizing SEC (Standardless Element Coefficient) factors for the semi-quantitative analysis. Four powdered samples (Table S4) after drying in room temperature were mounted on aluminum stubs using double-sided adhesive carbon discs and carbon-coated.

Attenuated Total Reflection Fourier Transform Infrared Spectroscopy (ATR-FTIR)

Attenuated total reflection-Fourier transform infrared (ATR-FTIR) spectra were recorded using the Nicolet iS10 Mid FT-IR (Thermo Scientific Inc.) spectrophotometer fitted with an ATR device with a diamond crystal plate. Measurement conditions included: spectral range 4000-400 cm^-1^, spectral resolution 4 cm^-1^, scan number of 32, Ge/KBr beam splitter, DLaTGS detector with dynamic interferometer justification. OMNIC 9 (Thermo Fisher Scientific Inc.) analytical software was applied. ATR-FTIR technique used in this study is an alternative method for FT-IR sample preparation where raw material is placed directly onto the diamond crystal for data acquisition. Each sample was scanned 25 times to obtain the averaged spectrum.

The ATR-FTIR method was applied due to its advantage over transmission technique. In the ATR-FTIR there is no need for using hygroscopic KBr pellets in sample preparation. Therefore, avoided are problems related to the KBr pellets preparation, i.e., the pressure impact, possible sample contamination and changes in clay mineral structure during moisture removal (e.g. Madejová and Komadel, 2001). Moreover, the ATR technique allows for the surface analysis of the investigated material.

X-ray powder diffraction (XRD)

XRD analyses were performed on powdered samples (<10 μm) using a PANalytical X’Pert Pro MPD -multipurpose diffractometer (Almelo, Holand), powered by a Philips PW3040/60 X-ray generator and fitted with a 1D silicon strip detector (X’Celerator). The measurements were performed using Cu Kα-radiation with a wavelength of 0.1541874 nm, an acceleration voltage of 40 kV, a current of 40 mA, and with 0.01 °2θ step sizes in the range of 3° - 75° 2θ and 300 s measurement time per step. The data obtained were processed using HighScore+ software and the ICSD database and PDF4+ ICDD database. All XRD analyses were performed at the Institute of Earth Sciences, University of Silesia, Sosnowiec.

**Table S4** Adsorbents analyzed by SEM

|  | Eu initial concentration in solution (mg/L) | pH |
| --- | --- | --- |
| Bentonite | 400 | 7 |
| Phyllite | 400 | 7 |
| 50%Bentonite+50%Phyllite | 400 | 7 |
| 50%Bentonite+50%Phyllite | 500 | 4.5 |

**Table S5** Europium and oxygen contents in the Eu-precipitates on bentonite and phyllite

| **Bentonite (pH 7)** | | | | | **Phyllite (pH 7)** | | | | |
| --- | --- | --- | --- | --- | --- | --- | --- | --- | --- |
| O | | Eu | | | O | | | Eu | |
| Weight% | Atomic% | | Weight% | Atomic% | Weight% | Atomic% | Weight% | | Atomic% |
| 30.08 | 80.34 | | 69.92 | 19.66 | 29.24 | 79.70 | 70.76 | | 20.30 |
| 29.64 | 80.00 | | 70.37 | 20.00 | 29.97 | 80.25 | 70.03 | | 19.75 |
| 33.83 | 82.92 | | 66.17 | 17.08 | 34.35 | 83.25 | 65.65 | | 16.75 |
| 29.91 | 80.21 | | 70.10 | 19.79 | 29.00 | 79.50 | 71.01 | | 20.50 |
| 30.34 | 80.53 | | 69.67 | 19.47 | 31.83 | 81.60 | 68.17 | | 18.40 |
| 32.25 | 81.89 | | 67.75 | 18.11 | 31.21 | 81.17 | 68.79 | | 18.83 |
| 29.04 | 79.54 | | 70.96 | 20.46 | 33.31 | 82.59 | 66.69 | | 17.41 |
| 29.46 | 79.87 | | 70.54 | 20.13 | 30.78 | 80.85 | 69.22 | | 19.15 |
| 29.35 | 79.78 | | 70.65 | 20.22 | 31.08 | 81.07 | 68.92 | | 18.93 |
| 33.06 | 82.43 | | 66.94 | 17.57 | 31.87 | 81.63 | 68.13 | | 18.37 |
| Medium: | Medium: | | Medium: | Medium: | Medium: | Medium: | Medium: | | Medium: |
| **30.70** | **80.75** | | **69.31** | **19.25** | **31.26** | **81.16** | **68.74** | | **18.84** |

**pH and pH_PZC_**

The pH of rock-water suspension was measured using a pH-meter equipped with a glass-combined electrode in deionized water at the suspension ratio of 1:10.

The point of zero net charge for adsorbents (pH_PZC_ defined as the pH at which the charge of the colloidal particles equals 0) was determined using the method of Lazarević et al. (2007). The point of zero charge of the rock samples was determined by batch equilibration technique and was found from a plot of Δ(pH_f_- pH_i_) vs. pH_i_. pH_i_ is pH of 0.1 mol/L KNO_3_ solutions adjusted to range from 3.5 to 10 (10 points) by adding HCl or KOH solution. The pH_f_ is final pH after adding rocks samples to each solution of electrolyte and filtered. The solution:rocks ratio was 500:1, shaking time 24 h.

The pHpzc of the samples was determined as intersection point of the curve with the axis pH_i_ on the graph of Δ(pH_f_- pH_i_)= f(pH_i_).

The samples were positively charged due to the surface protonation at solution pH lower than pH_PZC_ of the samples; whereas, they were negative charged due to the surface deprotonation at the solution pH higher than pH_PZC_ of the samples.

**Multi-way analysis of variance.**

Calculations were performed using the StatSoft STATISTICA and PQStat software. The Brown-Forsythe and Levene tests were used to study the equality of variances. The assumption of the equality of variance was met because p-values were lower than the level of significance α=0.05. The p-value was slightly higher (0.053083) only in the Brown-Forsythe test (Supplement Table S6). The assumption of the distribution normality was also met (Supplement Fig. S1).

The ANOVA results are given in the Supplement Fig. S2, S3, and Table S7. The mean values of *RE* (%) for various pH, L:S, Phy/B, and initial concentrations are shown in Table S5.

The interactions among variables (pH*L:S*Phy:B*C_O_) are not statistically significant (Supplement Fig. S3) because p-value (0.999955) is higher than the level of significance α=0.05 (Supplement Table S7). However, the different *RE* values obtained for pH 4.5 and 7 are statistically significant (p-value=0.000003 is lower than α=0.050). These differences occur regardless of the Eu(III) initial concentrations, L:S, and Phy:B. This applies also to other variables (Supplement Table S7). The differences in *RE* are not statistically significant (Supplement Table S8) for the mixtures with the Phy/B proportions of 75/25, 50/50 (p-value=0.103876), 25/75, and for B (p-value=0.17537),

The mean *RE* values were higher for pH 7 than 4.5 and were higher for L:S of 100:1 than for 500:1. The highest *RE* was achieved for *C_0_*=10 mg/L (Supplement Fig. S2, S3, and Table S9). The *RE* increased from the lowest mean value for phyllite, through 75Phy/25B and 50Phy/50B to the highest values for 25Phy/75B and B (Supplement Fig. S2, S3, and Table S9).

From the regression model (regression approach to ANOVA) and based on parameters of the model (Supplement Table S10) the *RE* values were 11.21% higher for pH 7 than for 4.5. The *RE* value was lower by 21.08% for L:S of 500:1 than of 100:1. The values of *RE* were lower by 5.32-45.23% for the initial concentration range of 25-200 mg/L compared to 10 mg/L. These differences are statistically significant, except for *C_0_*=25 mg/L (Supplement Table S10). Compared to the 25Phy/75B mixture, *RE* was lower by 5.87% and 20.7% for the 50Phy/50B and 75Phy/25B mixtures, respectively, and was lower by about 38.44% for Phy (Supplement Table S10).

**Table S6** Test of equality of variance

|  | Levene test | | Brown-Forsythe test | |
| --- | --- | --- | --- | --- |
|  | F | p | F | p |
| pH | 6.104 | 0.014916 | 3.818 | 0.053083 |
| Phy/B | 6.186 | 0.000152 | 5.264 | 0.000626 |
| L : S | 12.817 | 0.0005 | 14.850 | 0.00019 |
| C_0_ (mg/L) | 8.559 | 0.000001 | 6.968 | 0.00001 |

p - p values; F - F ratio

**Table S7** Result sheet of variance analysis

|  | SS | MS | DF | F | p |
| --- | --- | --- | --- | --- | --- |
| intercept | 688544.78 | 688544.78 | 1 | 4481.109 | <0.000001 |
| pH | 3766.90 | 3766.90 | 1 | 24.515 | 0.000003 |
| Phylite (Phy) / Bentonite (B) | 29906.81 | 7476.70 | 4 | 48.659 | <0.000001 |
| L : S | 13335.42 | 13335.42 | 1 | 86.788 | <0.000001 |
| C_0_ (mg/L) | 32221.46 | 6444.29 | 5 | 41.940 | <0.000001 |
| within(error) | 16594.74 | 153.66 | 108 |  |  |
| total | 95825.32 |  |  |  |  |
| pH*(Phy) /(B)*L : S*C_O_ |  |  |  | 0.185 | 0.999955 |
| pH*L : S |  |  |  | 0.056 | 0.813257 |
| pH*C_O_ |  |  |  | 0.119 | 0.98784 |
| pH*(Phy)/(B) |  |  |  | 0.641 | 0.63463 |
| L : S*(Phy)/(B) |  |  |  | 0.239 | 0.916023 |
| L : S*C_O_ |  |  |  | 1.714 | 0.137516 |
| (Phy)/(B)*CO |  |  |  | 0.864 | 0.630749 |

p - p values, F - F ratio, MS - Mean squares, SS - sum of squares, DF - degrees of freedom

**Table S8** Results of post-hoc test (Fisher test)

|  | p |
| --- | --- |
| pH 4.5 ; pH 7 | 0.000003 |
| Phy/B 25/75 ; Phy/B 50/50 | 0.103876 |
| Phy/B 25/75 ; Phy/B 75/25 | <0.000001 |
| Phy/B 25/75 ; B | 0.17537 |
| Phy/B 25/75 ; Phy | <0.000001 |
| Phy/B 50/50; Phy/B 75/25 | 0.000051 |
| Phy/B 50/50 ; B | 0.003309 |
| Phy/B 50/50 ; Phy | <0.000001 |
| Phy/B 75/25 ; B | <0.000001 |
| Phy/B 75/25 ; Phy | 0.000004 |
| B ; Phy | <0.000001 |
| L:S 100:1 ; L:S 500:1 | <0.000001 |
| 10 mg/L ; 25 mg/L | 0.177028 |
| 10 mg/L ; 50 mg/L | 0.001014 |
| 10 mg/L ; 100 mg/L | <0.000001 |
| 10 mg/L ; 150 mg/L | <0.000001 |
| 10 mg/L ; 200 mg/L | <0.000001 |
| 25 mg/L ; 50 mg/L | 0.04589 |
| 25 mg/L ; 100 mg/L | 0.000001 |
| 25 mg/L ; 150 mg/L | <0.000001 |
| 25 mg/L ; 200 mg/L | <0.000001 |
| 50 mg/L ; 100 mg/L | 0.001338 |
| 50 mg/L ; 150 mg/L | <0.000001 |
| 50 mg/L ; 200 mg/L | <0.000001 |
| 100 mg/L ; 150 mg/L | 0.007561 |
| 100 mg/L ; 200 mg/L | 0.000004 |
| 150 mg/L ; 200 mg/L | 0.034145 |

p - p values

**Table S9** Basic descriptive statistics

|  | n | X | S | standard error | -95% | 95% |
| --- | --- | --- | --- | --- | --- | --- |
| pH 4.5 | 60 | 70.15 | 30.69 | 3.962 | 62.219 | 78.073 |
| pH 7 | 60 | 81.35 | 24.87 | 3.211 | 74.926 | 87.777 |
| Phy/B = 25/75 | 24 | 87.83 | 19.44 | 3.967 | 79.621 | 96.035 |
| Phy/B=50/50 | 24 | 81.96 | 22.94 | 4.682 | 72.273 | 91.644 |
| Phy/B=75/25 | 24 | 66.86 | 28.73 | 5.864 | 54.733 | 78.992 |
| Bentonite | 24 | 92.71 | 14.80 | 3.021 | 86.459 | 98.959 |
| Phylite | 24 | 49.39 | 30.30 | 6.184 | 36.592 | 62.179 |
| L:S=100:1 | 60 | 86.29 | 22.22 | 2.869 | 80.550 | 92.031 |
| L:S=500:1 | 60 | 65.21 | 30.07 | 3.882 | 57.439 | 72.975 |
| 10 mg/L | 20 | 96.88 | 10.19 | 2.280 | 92.108 | 101.651 |
| 25 mg/L | 20 | 91.55 | 16.49 | 3.687 | 83.836 | 99.270 |
| 50 mg/L | 20 | 83.64 | 22.47 | 5.024 | 73.121 | 94.151 |
| 100 mg/L | 20 | 70.73 | 27.20 | 6.082 | 57.996 | 83.455 |
| 150 mg/L | 20 | 60.05 | 28.55 | 6.383 | 46.695 | 73.414 |
| 200 mg/L | 20 | 51.64 | 30.20 | 6.754 | 37.508 | 65.780 |

n - sample size. X - arithmetic mean. S - standard deviation

**Table S10** Model parametrs

|  | b | error of b | -95% | 95% | t | p |
| --- | --- | --- | --- | --- | --- | --- |
| intercept | 113.90 | 3.920 | 106.128 | 121.668 | 29.056 | <0.000001 |
| pH 4.5 - REF; pH 7 - [1] | | | | | | |
| pH [1] | 11.21 | 2.263 | 6.720 | 15.691 | 4.951 | 0.000003 |
| Phy/B 25/75 - REF; Phy/B 50/50 - [1]; Phy/B 75/25 - [2]; B - [3]; Phy - [4] | | | | | | |
| Phy/B[1] | -5.87 | 3.578 | -12.962 | 1.224 | -1.640 | 0.103876 |
| Phy/B[2] | -20.97 | 3.578 | -28.058 | -13.873 | -5.859 | <0.000001 |
| Phy/B[3] | 4.88 | 3.578 | -2.212 | 11.974 | 1.364 | 0.17537 |
| Phy/B[4] | -38.44 | 3.578 | -45.535 | -31.350 | -10.743 | <0.000001 |
| 100(L):1(S) - REF; 500(L):1(S) - [1] | | | | | | |
| L : S | -21.08 | 2.263 | -25.569 | -16.598 | -9.316 | <0.000001 |
| 10 mg/L - REF; 25 mg/L - [1]; 50 mg/L - [2]; 100 mg/L - [3]; 150 mg/L - [4]; 200 mg/L - [5] | | | | | | |
| C_0_ (mg/L)[1] | -5.33 | 3.920 | -13.096 | 2.443 | -1.359 | 0.177028 |
| C_0_ (mg/L)[2] | -13.24 | 3.920 | -21.013 | -5.474 | -3.379 | 0.001014 |
| C_0_ (mg/L)[3] | -26.15 | 3.920 | -33.924 | -18.384 | -6.672 | <0.000001 |
| C_0_ (mg/L)[4] | -36.83 | 3.920 | -44.595 | -29.055 | -9.394 | <0.000001 |
| C_0_ (mg/L)[5] | -45.24 | 3.920 | -53.005 | -37.466 | -11.540 | <0.000001 |

b - index of b; t - statistic of t; p - p values


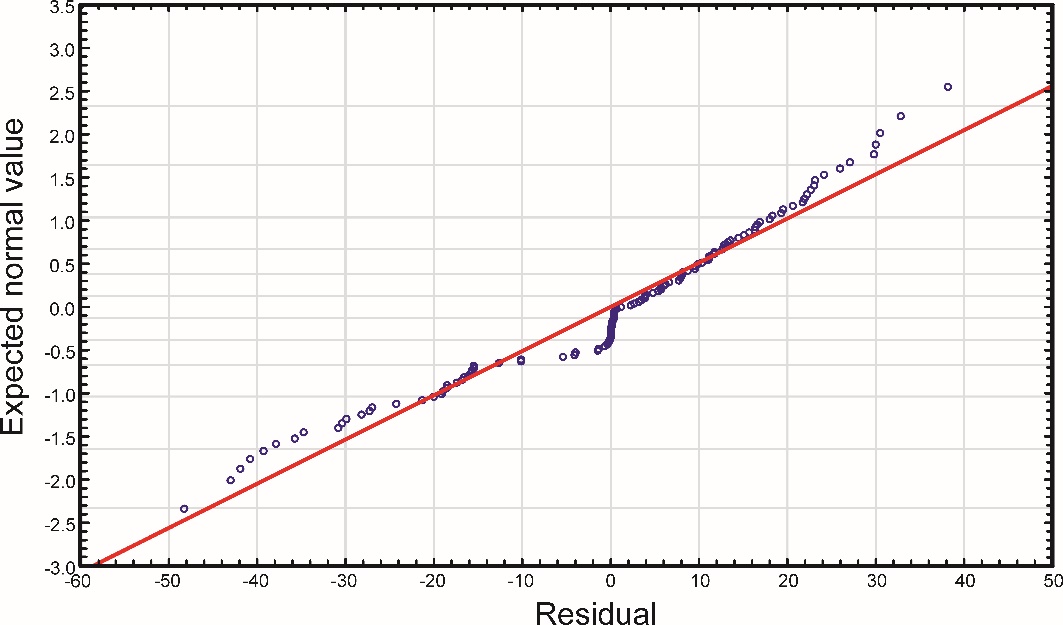


**Fig. S1** Normal Q-Q plot


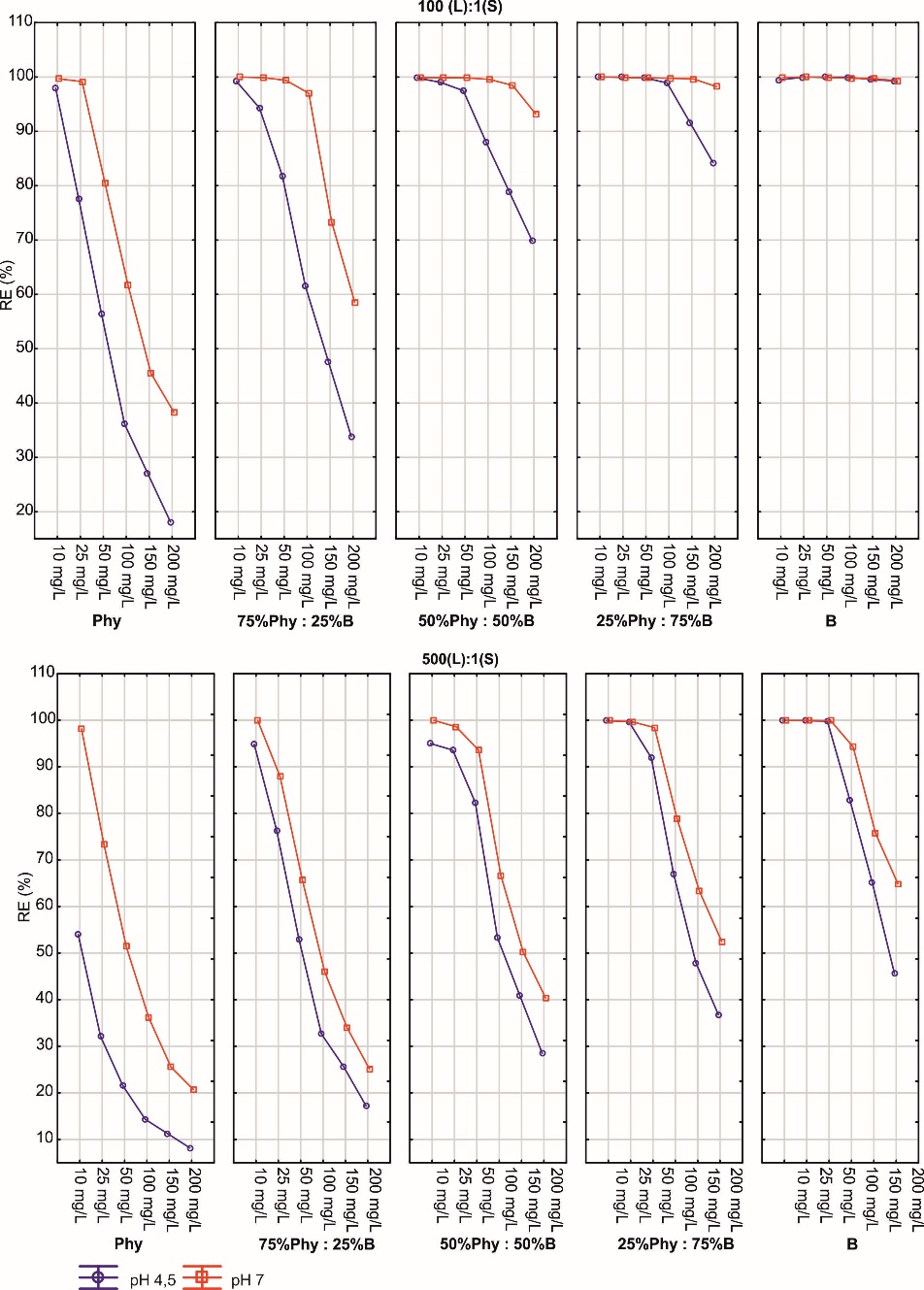


**Fig. S2** Plot of interaction for pH. L:S ratio. Phy/B and C_0_

_
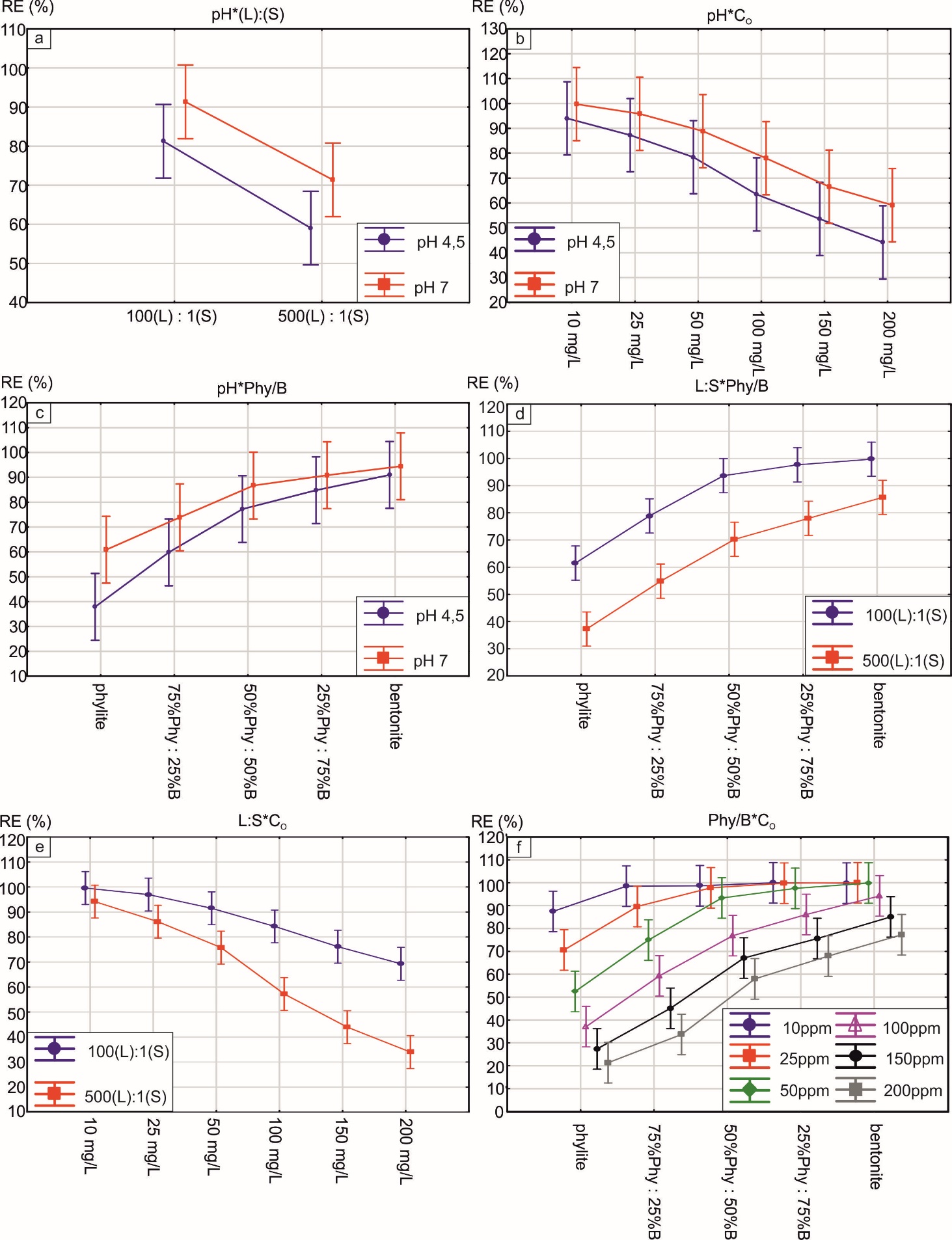
_

**Fig. S3** Graphs of the relationship between pH, L:S, C_0_, pH and Phy/B Madejová and Komadel, 2001, Carlson, 2004

**FT-IR interpretation**

Fig. S4 and S5 show the sets of FT-IR spectra of the phyllite and bentonite samples before and after europium adsorption at pH 4.5 and 7.0. Fig. S6 shows the spectra of mixtures of both these adsorbents, also before and after Eu adsorption and, for Comparison, FT-IR spectra of phyllite and bentonite after Eu adsorption at pH 4.5.

The low intensity of the stretching vibration in the range from 4000 to 3000 cm^-1^ (Fig. 1a) means a relatively low content of structural hydroxyl groups and water hydroxyl groups absorbing in this spectral range. Hence, it follows that these groups participation in the chemisorption of Eu^3+^ cations to the OH groups, also in the dissociated form, should also be small. Moreover, it follows that water participation in the formation of hydration shells around Eu^3+^ cations is small. The spectra shape of the phyllite samples indicates the probability of the processes mentioned above. After enlarging this range, a subtle structure of the spectra was revealed, showing a series of faint but separated bands (Fig. S4b). Comparison of the shape of these spectra indicates slight differences between them, which suggest a specific dependence of the spectra shape on pH and the nature of adsorption. Structural hydroxyl groups are present both inside and outside the packets. Weak bands at 3679 and 3652 cm^-1^ are attributed to vibrations of free silanol groups hydrogen-bonded to siloxane groups in the outer packet layer. The formation of hydrogen bonds on the surface of clay minerals is indicated by literature data (e.g., Johnston, 2010). The polarization of the resulting bonds may explain some mechanisms of cation sorption on these minerals. The range of hydrogen bonds is small here (as evidenced by relatively narrow bands), but the effect of these interactions may be the formation of active centers that are conducive to adsorption processes. From about 3560 to about 3500 cm^-1^, the spectra of the samples after adsorption show a similar shape, i.e., the adsorption does not clearly depend on pH. The hydrogen bonds can explain some mechanisms of Eu sorption on clay minerals (e.g., Bradbury and Baeyens, 2002). In the region under discussion, the band at 3622 cm^-1^ is ascribed to inner OH groups of the Al_2_OH fragment of mineral structure (e.g., Sdiri et al. 2016). A band at 3537cm^-1^ is due to stretching vibrations of the O-H bond in the SiAlO-OH group). The series of bands in the analyzed region indicate that some individual bands do not overlap here with bands of water bound with hydrogen bonds. This allows to identification of various hydroxyl groups which can be the centers of europium cations sorption. The overlapping band of water broadened by hydrogen bonds can be observed at about 3380cm^-1^.

There are no significant differences in spectra shapes in the range from ca. 1300 – 400 cm-1 (Fig.S4c). Some slight differences in the spectra shape after adsorption are noticeable in the range 600-890cm^-1^, especially in the case of the band at 750 cm^-1^, which comes from the libration vibrations of the (SiAl)O-OH group with a clear polar character (Shirozu, Ishida, 1982). Its partial dissociation could be the reason for the Eu(III) ion attachment.

Fig. S5 presents the spectra of bentonite before and after europium adsorption. Bentonite adsorbs significant amounts of water that dominates the spectra in the region: 3000-4000cm^-1^. Water probably blocks many of the sorption centers on the mineral surface. Simultaneously, water can cause Eu hydration, which is one of the immobilization manners of this element. The bentonite spectra show a similar shape for the sample before and after sorption. For samples after adsorption, this means a weak dependence on the pH of the adsorption capacity illustrated by IR spectra. The visible variation in the spectra shapes of the samples before and after adsorption, shown in the range of 3380-2500 cm^-1^, suggests the accumulation of larger amounts of water, probably bound by hydrogen bonds with polar groups binding Eu (e.g. Eu(OH)_2_^+^.

Fig. S4 FT-IR spectra of the phyllite samples before and after europium adsorption at pH 4.5 and 7.0. (a) in 400-4000cm^-1^, (b) 3300-3700 cm^-1^, (c) 400-1300 cm^-1^

Fig. S5 FT-IR spectra of the bentonite samples before and after Eu adsorption at pH 4.5 and 7.0. (a) in 400-4000cm^-1^, (b) 2700-3800 cm^-1^, (c) 400-870 cm^-1^

Fig. S6 FT-IR spectra of the Phyl/B mixtures, bentonite, and phyllite after Eu sorption (pH = 4.5) (a) in 400-4000cm^-1^, (b) 3300-3700 cm^-1^, (c) 400-1800 cm^-1^

Fig. S6 shows a comparison of the spectra of the initial Phy/B mixture after Eu adsorption and the individual components of the mixture after adsorption and under acidic pH conditions (pH = 4.5).

The tendency for spectra averaging is observed in the case of adsorbent mixtures. It is probably caused by interactions of a 1:1 mixture of mineral components, which may weaken or intensify individual adsorption processes due to physical and chemical interactions. Subtle but noticeable changes in the spectra are confirmed by the observations presented when discussing the spectra of the phyllite and bentonite groups, including the significant role of oxygen groups (mainly hydroxyl) and water molecules (hydration) in the Eu sorption process.

Bradbury M H, Baeyens. B (2022) Sorption of Eu on Na- and Ca-montmorillonites: Experimental investigations and modelling with cation exchange and surface complexation. Geochim Cosmoch Acta 66:2325–2334. https://doi.org/10.1016/S0016-7037(02)00841-4

Sdiri A, Khairy A, Bouaziz S, El-Satty S (2016) . A natural clayey adsorbent for selective removal of lead from aqueous solutions. Appl Clay Sci 26:89-97,
https://doi.org/10.1016/j.clay.2016.03.003

Johnston C T (2010) Probing the nanoscale architecture of clay minerals. Clay Miner 45(3):245-279. https://doi.org/10.1180/claymin.2010.045.3.245

Shirozu H, Ishida K (1982) Infrared study of some 7 and 14 Å layers. Miner J 11:161-171, https://doi.org/10.2465/minerj.11.161
